# Stalled ribosome rescue factors exert different roles depending on types of antibiotics in *Escherichia coli*

**DOI:** 10.1038/s44259-024-00039-2

**Published:** 2024-09-02

**Authors:** Mayu Mikami, Hidehiko Shimizu, Norika Iwama, Mihono Yajima, Kanako Kuwasako, Yoshitoshi Ogura, Hyouta Himeno, Daisuke Kurita, Nobukazu Nameki

**Affiliations:** 1 Division of Molecular Science, Graduate School of Science and Technology, Gunma University, 1-5-1 Tenjin-cho, Kiryu-shi, Gunma 376-8515 Japan; 2 Faculty of Pharmacy and Research Institute of Pharmaceutical Sciences, Musashino University, 1-1-20 Shinmachi, Nishitokyo-shi, Tokyo, 202-8585 Japan; 3 Division of Microbiology, Department of Infectious Medicine, Kurume University School of Medicine, Kurume, Fukuoka 830-0011 Japan; 4 Department of Biochemistry and Molecular Biology, Faculty of Agriculture and Life Science, Hirosaki University, Hirosaki, Japan

**Keywords:** Antimicrobial resistance, Ribosome

## Abstract

*Escherichia coli* possesses three stalled-ribosome rescue factors, tmRNA·SmpB (primary factor), ArfA (alternative factor to tmRNA·SmpB), and ArfB. Here, we examined the susceptibility of rescue factor-deficient strains from *E. coli* SE15 to various ribosome-targeting antibiotics. Aminoglycosides specifically decreased the growth of the Δ*ssrA* (tmRNA gene) strain, in which the levels of reactive oxygen species were elevated. The decrease in growth of Δ*ssrA* could not be complemented by plasmid-borne expression of *arfA*, *arfB*, or *ssrA*^*AA to DD*^ mutant gene possessing a proteolysis-resistant tag sequence. These results highlight the significance of tmRNA·SmpB-mediated proteolysis during growth under aminoglycoside stress. In contrast, tetracyclines or amphenicols decreased the growth of the Δ*arfA* strain despite the presence of tmRNA·SmpB. Quantitative RT-PCR revealed that tetracyclines and amphenicols, but not aminoglycosides, considerably induced mRNA expression of *arfA*. These findings indicate that tmRNA·SmpB, and ArfA exert differing functions during stalled-ribosome rescue depending on the type of ribosome-targeting antibiotic.

## Introduction

Despite non-stress conditions, ribosomes often become stalled while translating problematic mRNAs such as truncated or non-stop mRNAs generated for various reasons^1,2^. Consequently, such ribosomes are stalled at the 3′ end of the truncated mRNAs, leaving both the A-sites and mRNA entry channels of ribosomes vacant, and are referred to as non-stop ribosomes. Unless peptidyl-tRNA drop-off occurs during the early elongation stage of translation, rescuing non-stop ribosomes requires peptidyl-tRNA hydrolysis at the P-site of the ribosome, allowing the recycling of ribosomes. The means of rescuing non-stop ribosomes are classified into two types based on the ribosome state. The hydrolysis occurs in mRNA-bound ribosomes in one type, and in the large ribosomal subunit (one of the two subunits into which stalled ribosomes are split) in the other^3^. We focused on the former type, as the antibiotics used do not induce ribosome splitting. Bacteria contain one or more ribosome rescue factors that enter the empty A-sites of non-stop ribosomes. The primary conserved ribosome rescue factor is a ribonucleoprotein complex of tmRNA and SmpB (tmRNA·SmpB). Apart from a few exceptions (reviewed in refs. ^1,2,4,5^), most bacteria also possess either ArfA (formerly termed YhdL), ArfB (YaeJ), or both. However, the reason behind the combination of differing ribosome rescue factors depending on the bacterial type is unclear. Structural studies revealed that although the three ribosome rescue factors share common functionalities regarding the use of the C-terminal tail of each protein, the sequence and length differ^6^, and the molecular mechanisms for stalled-ribosome rescue vary considerably. These mechanisms are briefly described in the following sections.

The tmRNA·SmpB complex mediates a unique translation, where one protein is synthesized using two different mRNAs via *trans*-translation^7–9^. Initially, the tRNA-like domain of the tmRNA is aminoacylated with alanine by alanyl-tRNA synthetase. The alanylated tmRNA·SmpB bound to elongation factor EF-Tu enters the empty A site of a non-stop ribosome, and the structural domain of the bound SmpB acts as the tRNA anticodon stem-loop. The C-terminal tail enters a vacant mRNA entry channel of the ribosomal small subunit, where it partially adopts an α-helix conformation^10,11^. Following its release from EF-Tu, tmRNA·SmpB is accommodated at the A site. The nascent polypeptide from peptidyl-tRNA is transferred to the alanine moiety of alanyl-tmRNA, and translation switches the non-stop mRNA to the mRNA-like domain of tmRNA that possesses an internal open reading frame (ORF) encoding a tag peptide, which is 10 amino acids in the case of *E. coli*. The C-terminal tail is rotated and deeply inserted into the mRNA exit tunnel; ribosomes stop at the in-frame stop codon of the ORF; and class I polypeptide chain release factors 1 or 2 (RF1 or RF2) hydrolyzes the peptidyl-tRNA at the-P site. Accordingly, an 11-amino acid tag-peptide is added to the C-terminus of the nascent polypeptide. Notably, as the tag peptide acts as a recognition site for several cellular proteases such as ClpXP, ClpAP, Lon, FtsH, and Tsp^12^, the resulting tagged polypeptide, which is potentially deleterious to cells, is preferentially degraded by tag-specific proteases. Additionally, *trans*-translation allows for non-stop mRNA decay mediated by the 3′-to 5′-exoribonuclease RNase R to prevent further ribosome stalling events^13–15^. Thus, the tmRNA·SmpB rescue system facilitates quality control for both mRNAs and proteins.

A second ribosome rescue factor is ArfA that is phylogenetically restricted to a subset of γ-proteobacteria and *Neisseriaceae* (β-proteobacteria)^2^. Synthetic lethality screening in *Escherichia coli* revealed that the gene is essential for *E. coli* viability in the absence of tmRNA*-*coding *ssrA*. Thus, the gene was named alternative ribosome-rescue factor A (*arfA*)^16^. The prime function of ArfA is not as an enzyme but as a ribosome-associated scaffold protein that recruits a canonical release factor RF2, but not RF1, to a stalled ribosome^17,18^. When ArfA binds in the vicinity of the decoding region in the empty A-site of a non-stop ribosome, the C-terminal region enters a vacant mRNA entry channel^19–22^. RF2 binds to ArfA associated with the ribosome, with a large interaction interface between them, and the bound RF2 hydrolyzes peptidyl-tRNA at the P site to rescue stalled ribosomes. Notably, ArfA synthesis is regulated by tmRNA·SmpB^23,24^, a characteristic that is well conserved among species possessing *arfA*^25^. The *arfA* mRNA transcript possesses a stem-loop structure at the 3′ end and is specifically targeted for cleavage by RNase III, thus generating a non-stop mRNA coding for C-terminally truncated ArfA protein. A ribosome translates the non-stop mRNA and stalls, and the stalled ribosome is rescued by tmRNA·SmpB. Simultaneously, the tmRNA-mediated tag peptide joins the truncated ArfA protein, and the resulting tagged protein is preferentially degraded. Thus, the expression of *arfA* in cells necessitates a defective or overwhelmed *trans*-translation process. Only under such condition can the truncated ArfA be released from stalled ribosomes, likely by ArfA itself, which is already expressed (but at low levels), or by ArfB^23,24^. The resultant protein always lacks the 17–19 C-terminus amino acids but is properly functional and is hereafter referred to as active ArfA for simplicity. As active ArfA is expressed at low levels when the tmRNA·SmpB rescue system is in operation, the ArfA rescue system is considered a backup.

The third ribosome rescue factor is ArfB, and it is more widely distributed than is ArfA among bacteria with the exception of some phyla such as *Thermodesulfobacteria*, *Deinococcus-Thermus*, and *Firmicutes*^2,26^. Unlike tmRNA·SmpB or ArfA, ArfB is a self-acting enzyme that functions as a peptidyl-tRNA hydrolase for stalled-ribosome rescue^27–29^. When ArfB encounters a non-stop ribosome, the C-terminal tail enters the vacant mRNA entry channel of the ribosome, where it forms a 15-residue α-helix, and the catalytic domain followed by the tail is accommodated to the A-site to hydrolyze peptidyl-tRNA at the P-site^30^. Synthetic lethality screening revealed that the lethal phenotype of an *ssrA* and *arfA* double deletion mutant was suppressed by plasmid-encoded *arfB* overexpression but not by products derived from a genomic copy of *arfB*^28^. Consequently, ArfB is a poor substitute for tmRNA·SmpB in *E. coli*, unlike ArfA.

This study focused on ribosome-targeting antibiotics that can stall translating ribosomes via various mechanisms to inhibit protein synthesis. Sensitivity to certain ribosome-targeting antibiotics has been reported for *ssrA*-deletion strains of a few bacteria. For example, *E. coli* strain MG1655 lacking *ssrA* (Δ*ssrA*) is more sensitive than the wild-type to kanamycin (Kan), streptomycin (Str), and erythromycin (Ery) to a considerable extent and to chloramphenicol (Chl) to a moderate extent, but it is not sensitive to tetracycline (Tet)^31,32^. Δ*ssrA* from *Synechocystis* cells is more sensitive to Chl and Ery than the wild-type, but it is not sensitive to Tet (data regarding aminoglycosides are not available)^33^. These findings indicate that tmRNA-SmpB contributes substantially to bacterial protection against ribosome-targeting antibiotics. However, little is known regarding the involvement of other ribosome rescue factors in the context of protection against antibiotics. Moreover, the reason for the differences in Δ*ssrA* sensitivities among the different ribosome-targeting antibiotic classes is unclear.

Ribosome-targeting antibiotics are assumed to leave ribosomes stalled somewhere in the middle of mRNA, referred to as no-go ribosomes. However, the conversion of no-go to non-stop ribosomes is induced under various stress conditions, including stress caused by antibiotics with several pathways, and the resultant non-stop ribosomes are rescued by tmRNA·SmpB. For example, mRNA cleavage resulting in non-stop mRNA is caused by ribosome-dependent endoribonucleolytic toxins such as RelE and unknown endonucleolytic enzyme(s)^34–39^, or the ribosome-independent endoribonucleolytic toxin MazF^40,41^. Additionally, certain aminoglycosides cause a translational read-through of a stop codon in mRNA or frameshifting at a stop codon, thus resulting in ribosomes stalled at the mRNA 3′ end^32^. Recently, a comprehensive structural analysis of *E. coli* demonstrated that during ribosome collision (of an upstream ribosome with a stalled ribosome), the ribosome-dependent endoribonuclease SmrB is recruited to the disome to cleave mRNA at the 5′ boundary of the stalled ribosome, producing a non-stop ribosome^42^. One cause is ribosome-targeting antibiotics such as Ery. Thus, ribosome-targeting antibiotics, regardless of their type, may lead to generate non-stop ribosomes that are excellent tmRNA·SmpB targets; however, little information is available regarding ArfA or ArfB.

In the present study, we demonstrated that tmRNA·SmpB and ArfA play different roles in stalled-ribosomes rescue in *E. coli* depending on the types of ribosome-targeting antibiotics. The relationship between tmRNA·SmpB and ArfA is not alternative in terms of such stalled-ribosome rescue. Apparently, a combination of tmRNA·SmpB and ArfA provides bacterial protection against a range of ribosome-targeting antibiotics.

## Results

### Inhibitory effects of each antibiotic group on growth differ among the ribosome rescue factor-deficient strains

In this study, we used *E. coli* SE15, a human commensal bacterium isolated from the feces of an healthy adult^43^. SE15, despite lacking many virulence-related genes, belongs to the *E. coli* phylogenetic group B2 that includes many extraintestinal pathogenic *E. coli*. Among the seven typical phylogenetic groups in *E. coli*, group B is a genetically distant group from group A, to which K12 derivatives such as MG1655 belong^44^.

To examine whether all ribosome rescue factors are involved in growth in the presence of various ribosome-targeting antibiotics, we constructed three single-gene deletion strains from SE15 using a suicide plasmid-mediated genome editing system^45^. These strains included Δ*ssrA* (tmRNA-deficient strain), Δ*arfA*, and Δ*arfB*. For gene deletion design, a major portion of the target gene ORF was removed to create a short ORF (4–9 codons) (for the deletion design, see “Methods”). This design may be useful for minimizing the polar effect, that is, the effect of gene deletion on the expression of an adjacent gene^46^.

For growth measurements, all strains were aerobically cultivated at 37 °C for 5 or 8 h in LB medium supplemented with each antibiotic at the starting point. During the cultivation period, they appeared to be in the exponential growth phase (Supplementary Fig. 1) where protein synthesis, a fundamental process required for cell growth, occurs frequently. Therefore, it is conceivable that ribosome rescue factors are even more necessary during the exponential growth phase than during other phases. In fact, a *trans*-translation reaction mediated by tmRNA·SmpB was reported to occur with high frequency during its growth phase^47^. Growth of the strains was assessed based on the optical density (OD_600_) of the LB medium at 600 nm. Unless otherwise indicated, the concentration of each antibiotic in the LB medium was half that of the inhibitory concentration for the wild-type strain grown for 8 h. This concentration was defined as the IC_50_ value in this study. The IC_50_ values of all the antibiotics were determined by nonlinear regression analysis of the dose-dependent curves for the wild-type strain (Supplementary Fig. 2). The IC_50_ values are summarized in Supplementary Table 1.

**Fig. 1:**
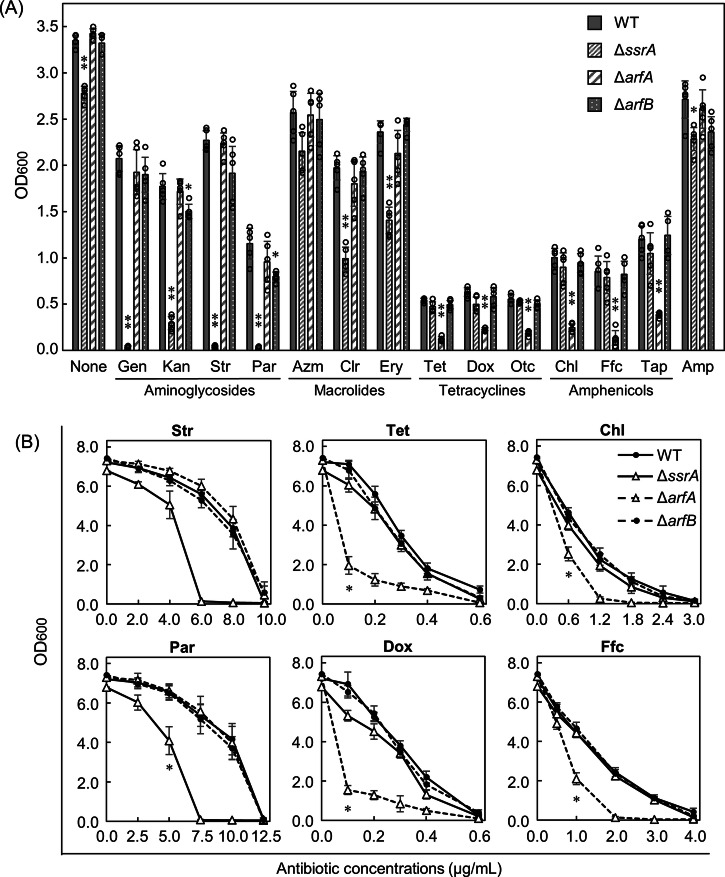
Comparison of inhibitory effects of ribosome-targeting antibiotics on growth among the wild-type and the three ribosome rescue factor-deficient strains. **A** Growth of the strains in the presence of various antibiotics. At the starting point, each strain was inoculated into 25 ml of LB medium at 37 °C in a 50 ml tube with a filter screw cap at a starting OD_600_ of 0.001. The LB medium contains each antibiotic at the IC_50_ concentration. After 5 h of growth in a shaking incubator, the OD_600_ of each culture was measured. It should be noted that the OD_600_ values of the wild-type strain after 5-h growth in the presence of each antibiotic at the IC_50_ concentration (particularly, tetracyclines and amphenicols) were not half of those in its absence, as the IC_50_ values were determined based on data after 8-h growth (Supplementary Fig. 2). Data are presented as the mean ± standard deviation of five independent experiments. Asterisks indicate significant differences compared to wild-type (Student’s *t* test, *p < 0.01; **p < 0.001). **B** Antibiotic concentration-dependent inhibition of growth of the wild-type and the three ribosome rescue factor-deficient strains. Each culture medium contained an antibiotic at the indicated concentration, and the OD_600_ of each medium was measured after 8 h of growth.

We used 13 ribosome-targeting antibiotics belonging to four classes, namely, aminoglycosides, tetracyclines, amphenicols, and macrolides. Although the antibiotic mechanism depends on the class, some delicate differences exist even among antibiotics of the same class. The fundamental mechanisms of action of the four antibiotic groups are briefly summarized in Table 1^48,49^. In the absence of antibiotics, the growth of Δ*arfA* or Δ*arfB* was comparable, whereas the growth of Δ*ssrA* slightly decreased compared to that of the wild-type strain (Fig. 1A). Similar results were reported for MG1655^50^. The most efficient growth under non-stress conditions apparently requires tmRNA·SmpB in *E. coli*.

**Table 1:** Summary of the fundamental mechanisms of the action of ribosome-targeting antibiotics used in the study

	Classes	Antibiotics	Abb.	Sub unit^a^	Binding sites	Mechanisms of action
Bactericidal	Aminoglycosides	Gentamicin	Gen	S	Helix 44 of 16S rRNA in the decoding center	Induce misreading of mRNA, resulting in the incorporation of incorrect amino acids into protein and/or inhibit translocation.
		Kanamycin	Kan			
		Streptomycin	Str			
		Paromomycin	Par			
Bacteriostatic	Tetracyclines	Tetracycline	Tet	S	Helix 34 of 16S rRNA	Inhibit aminoacyl-tRNA delivery into the A site by crashing the anticodon loop of the A-site tRNA.
		Doxycycline	Dox			
		Oxytetracycline	Otc			
	Amphenicols	Chloramphenicol	Chl	L	Peptidyl transferase center (PTC)	Inhibit peptide-bond formation by perturbing the correct positioning of the aminoacylated ends of tRNAs in the PTC.
		Florfenicol	Ffc			
		Thiamphenicol	Tap			
	Macrolides	Azithromycin	Azm	L	An rRNA pocket formed by domains II and V of 23S rRNA at the nascent peptide exit tunnel	Inhibit nascent chain elongation or block translocation by interacting with nascent peptides. Sequence-specific inhibition.
		Erythromycin	Ery			
		Clarithromycin	Clr			

^a^Each antibiotic binds to a small ribosomal subunit (S) or large ribosomal unit (L).

In the presence of the aminoglycosides Kan, gentamicin (Gen), Str, or paromomycin (Par), the growth of Δ*arfA* and Δ*arfB* was only slightly affected compared to that of the wild-type, whereas that of Δ*ssrA* was seriously impaired (Fig. 1A). Time-dependent growth measurements revealed that Δ*ssrA* was incapable of growing from the beginning in the presence of Str or Par (Supplementary Fig. 1). These results revealed that tmRNA·SmpB is indispensable for growth in the presence of aminoglycosides at the IC_50_ concentrations.

In the presence of the macrolides Ery and clarithromycin (Clr), the growth of Δ*ssrA* was reduced by only half compared to that of the wild-type, and the growth of Δ*arfA* and Δ*arfB* was similar to that of the wild-type (Fig. 1A). Azithromycin (Azm) exerted no significant effects on the growth of Δ*ssrA*, Δ*arfA*, or Δ*arfB*. Thus, among the ribosome rescue factors, tmRNA·SmpB rescued the stalled ribosomes during macrolide exposure most efficiently; however, its efficiency was lower than that during aminoglycoside exposure. This result indicated that ArfA can rescue the stalled ribosomes to some extent in place of tmRNA·SmpB.

Conversely, in the presence of the tetracyclines Tet, doxycycline (Dox), or oxytetracycline (Otc), the growth of Δ*arfA*, but not of Δ*ssrA* or Δ*arfB*, was reduced by approximately 70% (Fig. 1A). Similar results were observed in response to amphenicol, Chl, florfenicol (Ffc), and thiamphenicol (Tap) (Fig. 1A). The results in the presence of Tet or Chl were confirmed using time-dependent growth curves (Supplementary Fig. 1). Thus, despite the presence of tmRNA·SmpB, ArfA was involved in efficient growth in the presence of tetracyclines or amphenicols.

Additionally, in the presence of ampicillin (Amp) that does not target ribosomes, the growth of Δ*ssrA* but not of Δ*ssrA* or Δ*arfB* was slightly but significantly reduced by approximately 16% compared to that of the wild-type (Fig. 1A). This result is consistent with the results of a previous study demonstrating the sensitivity of Δ*ssrA* from MG1655 to Amp, although its sensitivity to SE15 appears to be lower than that of MG1655^31^. It is unclear why *ΔssrA* is sensitive to Amp, which is an inhibitor of cell wall synthesis. However, the absence of a particular sensitivity to Amp in the SE15 strains allowed Amp to be used for plasmid retention in subsequent experiments.

We assessed whether Δ*smpB* exhibited similar antibiotic sensitivities to those of Δ*ssrA*, as the ribosome rescue factor becomes functional only when tmRNA and SmpB form a complex. The results for Δ*smpB* were similar to those for Δ*ssrA* (Supplementary Fig. 3). Thus, growth in the presence of specific ribosome-targeting antibiotics requires both tmRNA and SmpB, or equivalently, their complex.

Next, we confirmed the dose-dependence of Str, Par, Tet, Dox, Chl, and Ffc on the growth of each ribosome rescue factor-deficient strain. Consequently, Δ*ssrA* was more sensitive to the aminoglycosides Str and Par than were Δ*arfA* or Δ*arfB* (Fig. 1B). Specifically, Δ*ssrA* could not grow in the presence of Str and Par above concentrations of 6.0 and 7.5 µg/ml, respectively. In contrast, Δ*arfA* was significantly more sensitive to the tetracyclines Tet and Dox and the amphenicols Chl and Ffc than were Δ*ssrA* or Δ*arfB* (Fig. 1B). These results were consistent with our previous findings.

Then, we assessed if the OD_600_ values of the wild-type and the three mutant strains grown in the presence of each antibiotic at IC_50_ concentrations reflected the number of live cells or both live and dead cells. Bacterial viability assays using 5-cyano-2,3-ditolyl tetrazolium chloride (CTC) (a fluorescent redox dye used to determine the respiratory activity of bacteria) and 4’,6-diamidino-2-phenylindole, dihydrochloride (DAPI) (a fluorescent DNA dye) revealed that most cells in the cultures after 5-h growth in the presence of Str, Par, Tet, or Chl at each IC_50_ concentration were alive regardless of the strain types, with the exception of Δ*ssrA*, in the presence of the aminoglycosides (Supplementary Fig. 4). Therefore, the OD_600_ values measured in the presence of an antibiotic appear to reflect the number of live cells.

Finally, to confirm these results, we examined antibiotic susceptibility using the broth microdiffusion method^51^ in which the minimum inhibitory concentrations (MICs) of each antibiotic were determined for the five strains (Supplementary Fig. 5). The MIC is the lowest concentration of an antibiotic that inhibits the visible growth of a microorganism after incubation for a defined period (16–20 h) or until satisfactory growth is obtained, and it defines the in vitro levels of susceptibility or resistance of a microorganism to an antibiotic. The MIC values of the five antibiotics in LB medium are presented in Table 2. The MICs of Str and Par against *ΔssrA* were half those against the wild-type, *ΔarfA* and *ΔarfB* strains. In contrast, the MICs of Tet, Dox and Chl against *ΔarfA* were half those of the wild-type, *ΔssrA* and *ΔarfB*. These results demonstrated that *ΔssrA* and *ΔarfA* are sensitive to the aminoglycosides and the tetracyclines/Chl, respectively, and are consistent with the results obtained in the above experiments.

**Table 2:** MICs of the antibiotics against wild type, Δ*ssrA*, Δ*arfA*, and Δ*arfB* from *E. coli* SE15

Antibiotics	MIC (µg/ml)			
	WT	Δ*ssrA*	Δ*arfA*	Δ*arfB*
Str	20	10	20	20
Par	20	10	20	20
Tet	1	1	0.5	1
Dox	2	2	1	2
Chl	4	4	2	4

For *E. coli* ATCC 25922, MICs were reported as follows^88^: Str, 8 µg/ml; Tet, 2 µg/ml; Dox, 1 µg/ml; Chl, 4 µg/ml.

Collectively, these findings reveal differences between tmRNA·SmpB and ArfA regarding the types of stalled-ribosome rescue depending on the antibiotic groups. Thus, ArfB is largely not involved in the rescue of antibiotic-dependent stalled ribosomes.

### Tet- or Chl-dependent growth reduction of Δ*arfA* can be restored by plasmid-borne expression of *ssrA*/*smpB* or *arfB*

**Fig. 2:**
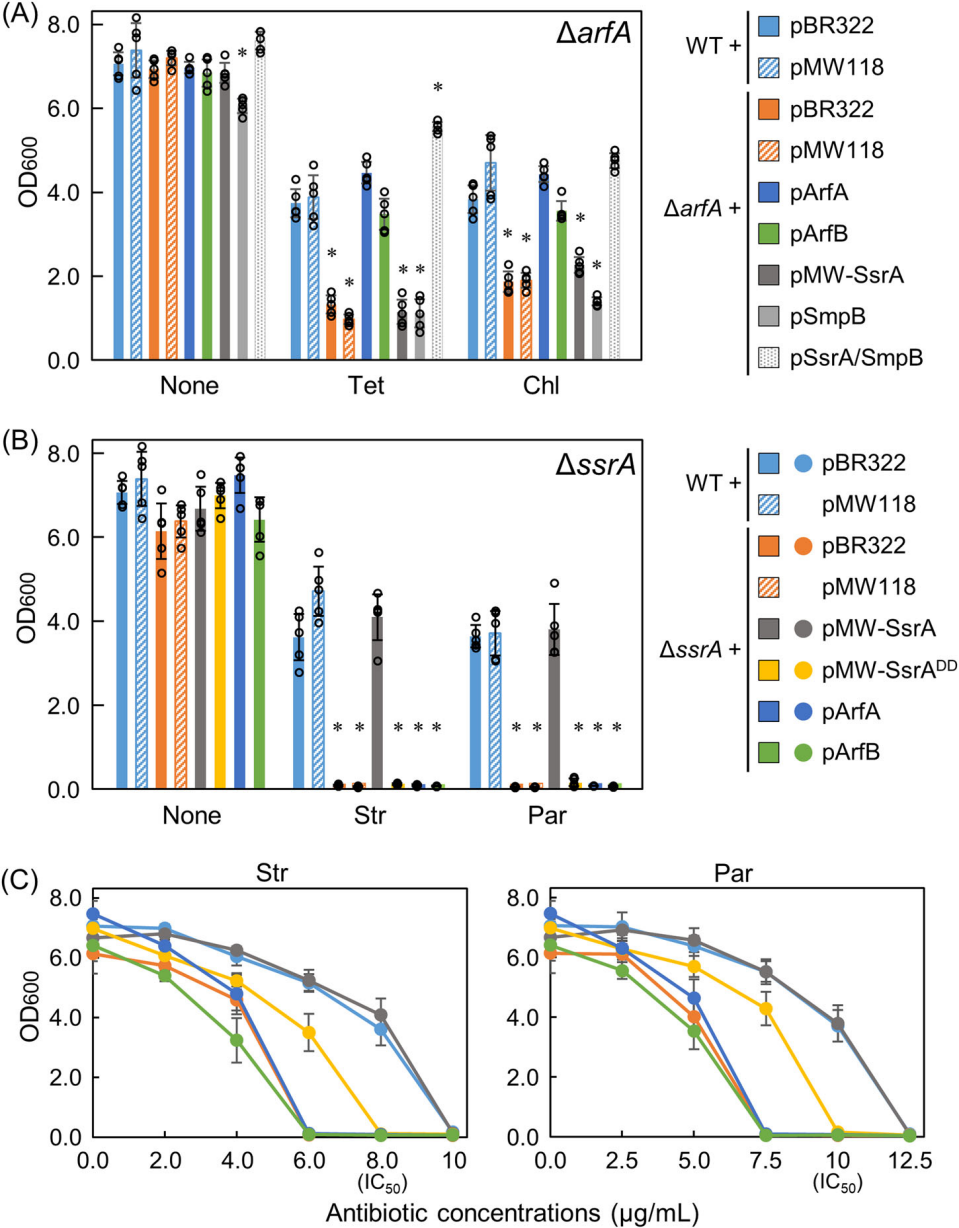
Suppression of the antibiotic susceptibility phenotypes of Δ*arfA* and Δ*ssrA* by a plasmid-borne ribosome rescue factor. **A** Δ*arfA* was transformed with a derivative of plasmid pBR322 harboring *arfA* (pArfA), *arfB* (pArfB), *smpB* (pSmpB), or both *ssrA* and *smpB* (pSsrA/SmpB) or that of plasmid pMW118 harboring *ssrA* (pMW-SsrA). As the controls, the wild-type and Δ*arfA* strains were transformed with an empty plasmid pBR322 or pMW118. In all experiments, the LB medium contains not only each indicted antibiotic at the IC_50_ concentration but also ampicillin for plasmid maintenance (pBR322, 50 µg/ml; pMW118, 12.5 µg/ml). The OD_600_ value of each medium was measured after 8 h of growth. Data are presented as the mean ± standard deviation of five independent experiments. Asterisks indicate significant differences compared to wild-type (Student’s *t* test, *p < 0.001). **B** Δ*ssrA* was transformed with pMW-SsrA, pMW-SsrA^DD^, pArfA, or pArfB. As the controls, the wild-type and Δ*ssrA* strains were transformed with an empty plasmid pBR322 or pMW118. **C** Str- and Par-concentration-dependent inhibition of growth of the transformant used in (**B**). The symbol legend is indicted in (**B**). For the control, the data for pMW118 are not shown in the graphs, as they were virtually identical to those for pBR322. Data are presented as the mean ± standard deviation of five independent experiments.

Other factors were expressed in each ribosomal rescue factor-deficient strain. A question arose regarding whether the major reason for growth reduction of Δ*arfA* in the presence of Tet or Chl was the lack of an intrinsic capability of ArfA to rescue such antibiotic-dependent stalled ribosomes or lack of expression levels of ArfA in cells. To address this question, we examined the recovery of the antibiotic-dependent growth reduction of Δ*arfA* at the IC_50_ concentration by plasmid-borne expression of other ribosome rescue factors. First, we examined whether Δ*arfA* growth reduction in the presence of Tet or Chl was only caused by the deletion of *arfA*. We transformed Δ*arfA* with a derivative of plasmid pBR322 containing partial-length ORF (corresponding to 54 amino acids out of 72) of *arfA* and the 200-bp region upstream of the ORF (termed pArfA). The mRNA transcribed from the truncated ORF was unable to form a stem loop; thus, the expressed ArfA protein was active^23,24^. Therefore, the Tet- or Chl-dependent growth reduction of Δ*arfA* was completely complemented by plasmid-borne expression of *arfA* (Fig. 2A). This result demonstrates that the Tet- or Chl-dependent growth reduction is attributable exclusively to *arfA* deletion.

Next, we examined whether Δ*arfA* growth reduction was complemented by the plasmid-borne expression of *arfB*. For the *arfB* expression plasmid (pArfB), the ORF of *arfA* in pArfA was replaced with that of *arfB* such that the expression levels of ArfA and ArfB were similar. Δ*arfA* growth reduction was complemented by plasmid-borne expression of *arfB* (Fig. 2A). Moreover, we examined whether the growth reduction of Δ*arfA* was complemented by plasmid-borne expression of *ssrA* or *smpB*. The plasmids for the expression of *ssrA* (pMW-SsrA) and *smpB* (pSmpB) were derivatives of pMW118 and pBR322, respectively (for the construction design, see “Methods”). However, the growth reduction of Δ*arfA* was not complemented by either plasmid (Fig. 2A). This result was sensible as tmRNA and SmpB function only when they are complexed; the increase in either would not lead to an increase in the tmRNA·SmpB complex in the cells. Therefore, we constructed a plasmid that coexpressed *ssrA* and *smpB*. Because these genes are located next to each other in *E. coli*, the entire region, ranging from *smpB* to *ssrA* was inserted into pBR322, based on a previous report^52^. Consequently, the Tet- or Chl- dependent growth reduction of Δ*arfA* was well complemented by plasmid-borne co-expression of *ssrA* and *smpB* (Fig. 2A).

Thus, the Tet- or Chl- dependent stalled ribosomes in Δ*arfA* could be rescued for efficient growth by tmRNA·SmpB or ArfB if its quantity in cells is increased. ArfB belongs to a protein group that is expressed at low levels in cells (for example, SmpB:ArfB = 1934:73 units of molecules per generation during ribosome profiling)^53^. Hence, the failure of ArfB to rescue those stalled ribosomes in Δ*arfA* could be attributable to the deficit of the protein in cells rather than to a lack of rescue ability. The reason underlying the observations for tmRNA·SmpB, which is apparently abundant in cells, is described in the “Discussion” section.

### Aminoglycoside-dependent growth reduction of Δ*ssrA* failed to be complemented by *arfA*, *arfB*, or *ssrA*^*DD*^

We examined whether the severe growth reduction of Δ*ssrA* in the presence of Str or Par at the IC_50_ concentration was complemented by plasmid-borne expression of *ssrA*, *arfA*, or *arfB*. The plasmids used were the same as those used in the previous experiments. The severe growth reduction of Δ*ssrA* was well complemented by plasmid-borne expression of *ssrA*, thus confirming that its reduction resulted solely from *ssrA* deletion (Fig. 2B). Conversely, the growth reduction of Δ*ssrA* was not complemented by plasmid-borne expression of *arfA* or *arfB*. As there are no proteolysis pathways specific to the ArfA or ArfB recue system, this result indicated that tag-dependent proteolysis of incomplete proteins contained in the tmRNA·SmpB rescue system is involved in Δ*ssrA* growth recovery.

To clarify whether the growth reduction of Δ*ssrA* is attributable to the absence of the tag-dependent proteolysis of incomplete proteins, we constructed a mutant derivative of pMW-SsrA in which the last two amino acid residues of the peptide tag (ANDENYALAA) were changed from Ala-Ala to Asp-Asp (termed pMW-SsrA^DD^). This tmRNA mutant is capable of rescuing stalled ribosomes and tagging target proteins with a variant tag. However, the resulting released incomplete proteins cannot be degraded rapidly^7,47,54,55^. The growth reduction of Δ*ssrA* by the addition of Str or Par at the IC_50_ concentration was not complemented by plasmid-borne expression of *ssrA*^*DD*^ (Fig. 2B). This result indicated the need for tag-dependent proteolysis of incomplete proteins for growth in the presence of aminoglycosides at each IC_50_ concentration. Similar results have been reported for *Streptomyces coelicolor*^56^. Spotting assay results demonstrated that growth of *S. coelicolor* Δ*ssrA* is severely inhibited by a sublethal concentration of the aminoglycoside hygromycin (5 µg/ml), and the reduced growth is restored by plasmid-borne expression of *ssrA* but not of *ssrA*^*DD*^.

Furthermore, we examined concentration-dependent effects of Str and Par on growth of the Δ*ssrA* strain transformed with pArfA, pArfB, pMW-SsrA, or pMW-SsrA^DD^. Over a wide range of concentrations of either antibiotic, Δ*ssrA* transformed with pMW-SsrA was virtually identical in growth to the wild-type strain transformed with an empty plasmid pBR322 or pMW118 (the control) (Fig. 2C). However, significant differences in sensitivities to Str and Par between the control and the other transformants were observed depending on the antibiotic concentration. In the presence of Str, growth of Δ*ssrA* transformed with pBR322, pArfA, or pArfB decreased in a concentration-dependent manner compared to that of the control, and these decreasing curves were similar among the three transformants (Fig. 2C). At a concentration of 6.0 µg/ml, no growth was observed for the three transformants, while growth of Δ*ssrA* transformed with pMW-SsrA^DD^ was approximately 70% of that of the control. At a concentration of 8.0 µg/ml (IC_50_), no growth was observed for the pMW-SsrA^DD^ transformant. Similar results were observed in the presence of Par (Fig. 2C).

Collectively, neither overexpression of *arfA* nor *arfB* improved the growth reduction of Δ*ssrA* in the presence of Str or Par at any concentration. In contrast, overexpression of *ssrA*^*DD*^ partially improved the growth reduction at Str and Par concentrations ranging from 6.0 µg/ml to 8.0 µg/ml and from 7.5 µg/ml to 10.0 µg/ml, respectively. Thus, the results indicated that aminoglycoside-dependent stalled ribosomes can hardly be rescued by ArfA or ArfB but can be partially rescued by the tmRNA·SmpB variant lacking tag-dependent proteolysis. It follows that tag-dependent proteolysis mediated by tmRNA·SmpB becomes increasingly important for growth in the presence of the aminoglycosides at concentrations above the IC_50_.

### Lack of tag-specific proteolysis increases ROS levels induced by aminoglycosides

Several lines of evidence indicate that bactericidal antibiotics such as aminoglycosides, ß-lactams, and fluoroquinolones elicit the production of reactive oxidants, including ROS^57,58^. ROS generation is also induced by ribosome-targeting antibiotics that are apparently linked to aberrant protein production^59–61^. Hence, we expected that aminoglycosides would increase intracellular levels of ROS more in Δ*ssrA* than in the wild-type strain because Δ*ssrA* lacked the tag-specific proteolysis pathway. To confirm this hypothesis, we examined intracellular ROS production in the ribosome rescue factor-deficient strains exposed to Str, Par, or Tet using the ROS assay kit consisting of a photo-oxidation resistant derivative of 2′,7′-dichlorodihydrofluorescein diacetate (DCFH-DA). After growing the strains for 2.5 h (OD_600_ of approximately 0.3), antibiotics were added at 1.5-fold the IC_50_ concentration to the media. The reason for selecting this concentration is described later. After the cultures were incubated for 3 h, the LB medium was replaced with PBS, and the oxidant-sensing probe was added according to the manufacturer’s protocol.

**Fig. 3:**
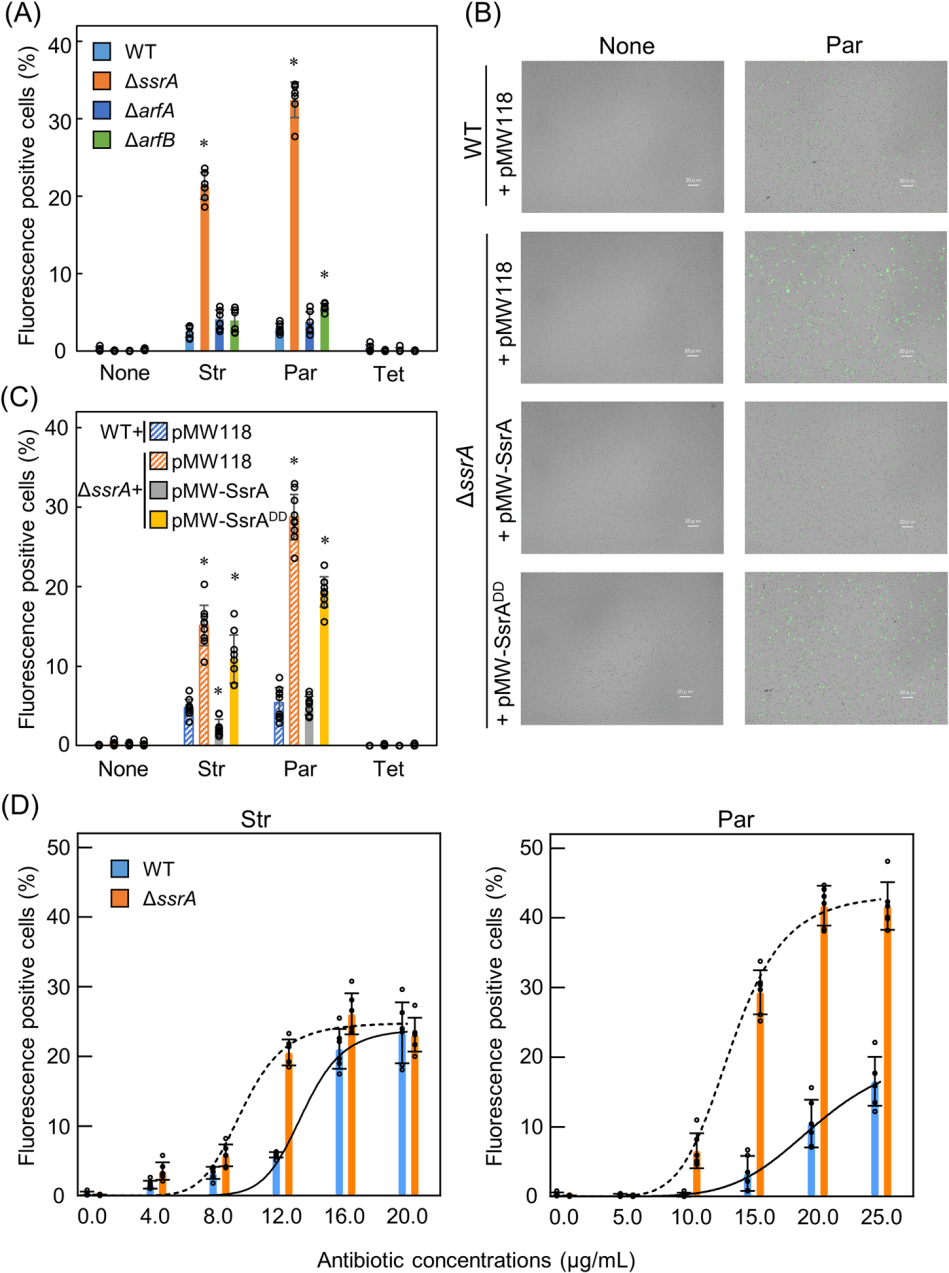
Significant ROS generation induced by the ribosome-targeting antibiotics in Δ*ssrA.* **A** Percentages of fluorescent-positive cells of antibiotic-treated wild-type, Δ*ssrA*, Δ*arfA*, and Δ*arfB* strains. After the strains were grown for 2.5 h, Str at 1.5-fold the IC_50_ concentrations (12.2 µg/ml), Par (14.6 µg/ml), or Tet (0.48 µg/ml) was added to the media, and they were further incubated for 3 h. The strain samples were stained with a photo-oxidation resistant derivative of DCFH-DA, and were adjusted to OD_600_ values of 2.0. All images were obtained by a fluorescence microscope (magnification, ×40; scale bar: 20 µm). Three fields per sample were analyzed, and each time, at least 100 cells were counted. Two independent experiments were performed for the wild-type and the mutant strains (*n* = 6). The results were expressed as percentage of fluorescent-positive cells versus cells observed in bright-field images. Data are presented as the mean ± standard deviation of the six data. Asterisks indicate significant difference compared to wild-type (Student’s *t* test, *p < 0.001). Representative merged bright-field and fluorescent microscopy images are presented in Supplementary Fig. 6A. **B** Representative merged bright-field and fluorescent microscopy images of antibiotic-treated cells of the wild-type and Δ*ssrA* strains transformed with an empty plasmid, pMW-SsrA, or pMW-SsrA^DD^ that were stained with the oxidant-sensing probe. Only representative images regarding non-antibiotic- and Par-treated cells are presented. The other images are presented in Supplementary Fig. 6B. **C** Percentages of fluorescent-positive cells of the antibiotic-treated transformants of the wild-type and Δ*ssrA*. For all others, refer to the legend in (**A**). **D** Percentages of fluorescent-positive cells of antibiotic-treated wild-type and Δ*ssrA* depending on the antibiotic concentrations. The variable slope sigmoidal dose-response best-fit curves generated in GraphPad Prism 9.3.1 are plotted (*R*^2^: WT [0.933 and 0.901] and Δ*ssrA* [0.944 and 0.984] for Str and Par, respectively). The curves for the wild-type and Δ*ssrA* are indicated by solid and dotted lines, respectively.

Bright-field and fluorescence images of the samples were captured using a fluorescence microscope, and the cells in each image were counted automatically using ImageJ2/Fiji^62^. The percentage of fluorescence-positive cells in each sample was determined as the ratio of the number of fluorescence-positive cells in the fluorescence image to the number of cells in the corresponding bright-field image. In the absence of antibiotics, none or very few fluorescent-positive cells representing ROS generation status were observed in all the four strains, namely, the wild-type, Δ*ssrA*, Δ*arfA*, and Δ*arfB* strains (Fig. 3A and Supplementary Fig. 6A). Upon exposure to Str or Par but not to Tet, the percentage of fluorescent-positive cells considerably increased in Δ*ssrA* compared to those in the wild-type, Δ*arfA*, or Δ*arfB* strains. This result suggested that tmRNA·SmpB suppressed the increase of ROS generation induced by the aminoglycosides.

To verify whether the increased levels of ROS in the presence of the aminoglycosides were because of the absence of tag-specific proteolysis mediated by tmRNA·SmpB, we used Δ*ssrA* strains transformed with the empty plasmid pMW118, pMW-SsrA, or pMW-SsrA^DD^. In the absence of antibiotics, none or fewer fluorescent cells were observed in all four transformed strains (Fig. 3B, C and Supplementary Fig. 6B). Upon exposure to Str and Par, fluorescent-positive cells of Δ*ssrA* transformed with the empty plasmid significantly increased in percentage by approximately four-fold compared to those of the wild-type strain with the empty plasmid. This result agreed well with the above result using the strains that were not transformed with a plasmid. In contrast, fluorescent-positive cells of Δ*ssrA* transformed with pMW-SsrA decreased to the level of those of the wild-type strain with the empty plasmid, thus confirming that elevated ROS levels in Δ*ssrA* were suppressed by plasmid-borne tmRNA. Conversely, fluorescent-positive cells of Δ*ssrA* transformed with pMW-SsrA^DD^ were similar in percentage to those of Δ*ssrA* with the empty plasmid, thus revealing that elevated ROS levels in Δ*ssrA* were not suppressed by plasmid-borne tmRNA variant. Thus, the lack of tag-specific proteolysis mediated by tmRNA·SmpB resulted in significant increase in intracellular ROS levels.

Finally, we examined antibiotic concentration-dependent ROS generation in the wild-type and Δ*ssrA* strains. Upon exposure to Str, the percentage of fluorescent-positive cells increased in the wild-type and Δ*ssrA* in a concentration-dependent manner, although this increase occurred at different rates (Fig. 3D and Supplementary Fig. 7). Notably, the plot of antibiotic concentrations versus the percentage of fluorescent-positive cells exhibited a nearly sigmoid curve irrespective of the type of strain, thus indicating that ROS were produced in large quantities when the antibiotic concentrations exceeded a certain level that almost corresponded to 1.5-fold the IC_50_ concentration. Furthermore, the results demonstrated that ROS generation in Δ*ssrA* occurred at a lower concentration than that in the wild-type strain. Similar results were obtained for Par, although Δ*ssrA* was even more sensitive to Par than to Str (Fig. 3D and Supplementary Fig. 7). It is most likely that these results are connected to those obtained from the previously mentioned experiments that demonstrated that above certain concentrations of aminoglycosides, tag-dependent proteolysis mediated by tmRNA·SmpB is required for growth.

### Expression of *arfA* mRNA is induced by tetracyclines and amphenicols

Adaptive responses to stresses, including that caused by antibiotics, induce the expression of specific genes as a bacterial survival strategy^57,63,64^. To determine whether the antibiotics exerted any effect on the transcription levels of each ribosome rescue factor gene, we performed quantitative reverse transcription PCR (qPCR). The wild-type SE15 strain was grown in the absence of antibiotics according to the procedure described above. When the culture medium reached an OD_600_ of 0.3 ~ 0.4, each antibiotic at double the IC_50_ concentration was added to the medium, and cultivation was continued for 30 min. Total RNA was extracted from the harvested samples. Based on the reliable reference genes in *E. coli* as identified by Zhou et al.^65^, *idnT* was selected as the reference gene in this study. We confirmed that the Ct value per amount of input mRNA remained largely unchanged no matter which of the eight antibiotics was added (Str, Par, Azm, Ery, Tet, Dox, Chl, and Ffc) (Supplementary Fig. 8).

**Fig. 4:**
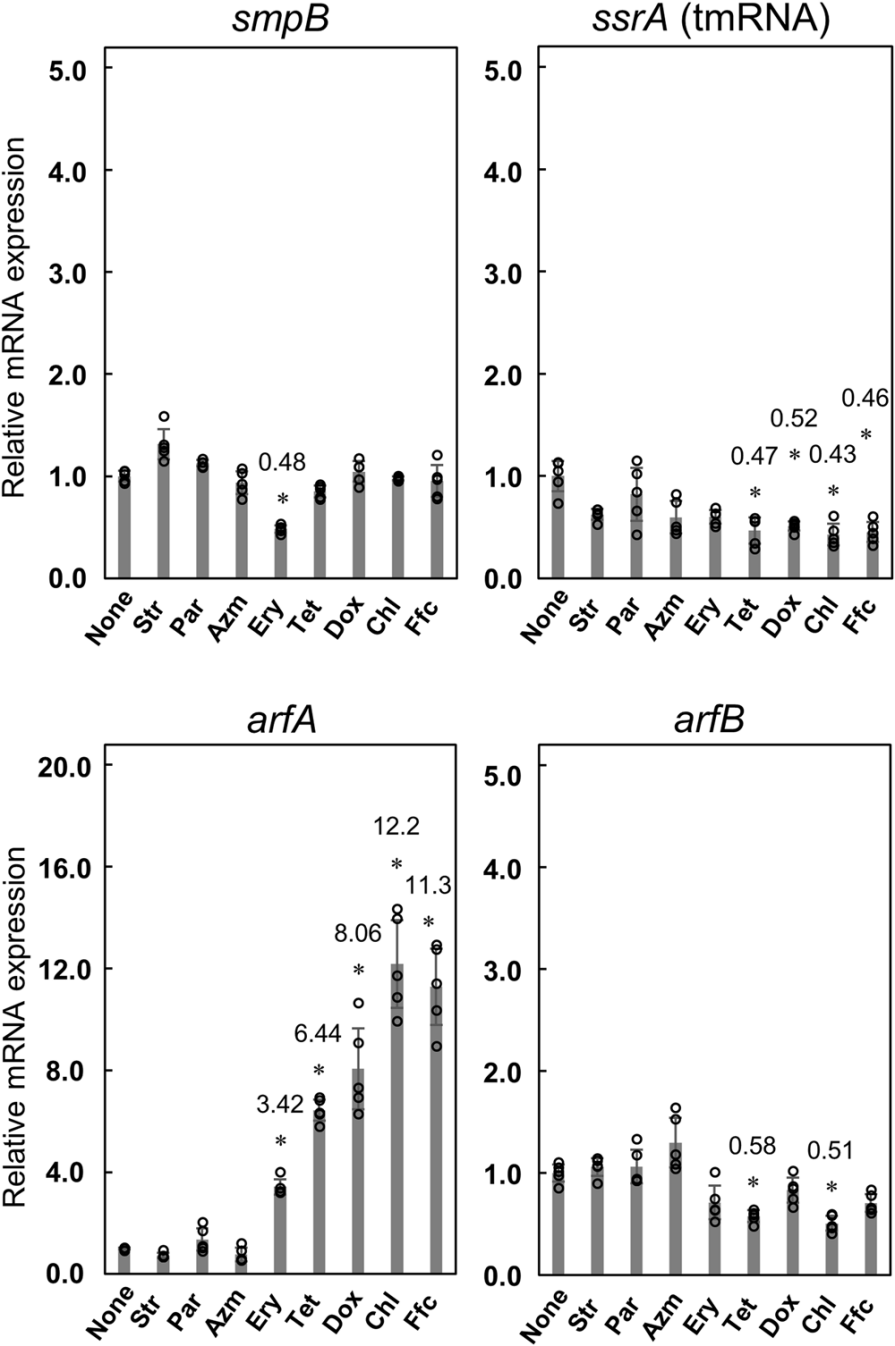
Effects of antibiotics on mRNA expression of *smpB*, *ssrA*, *arfA*, and *arfB* from total RNA extracted from the wild-type SE15 strain. When the culture medium reached an OD_600_ of 0.3 ~ 0.4, the indicated antibiotic at double the IC_50_ concentration was added to the medium. The samples were incubated for 30 min. *idnT* was used as an internal reference gene (see details in the text). Relative target gene expression levels were calculated using the equation 2^−ΔΔCt^, where ΔΔCt = ((Ct^antibiotic^_*target*_ – Ct^antibiotic^_*idnT*_) – (Ct^nontreated^_*target*_ – Ct^nontreated^_*idnT*_)). All Ct values used in the calculations are presented in Supplementary Fig. 8. Data are presented as the mean ± standard deviation of five independent experiments. Asterisks indicate significant differences compared to wild-type (Student’s *t* test, *p < 0.001). Values are described above the bars marked with asterisks.

Neither the mRNA expression levels of *smpB* nor *arfB* were substantially affected by the addition of antibiotics, whereas the *ssrA* expression level was reduced by approximately 50% upon the addition of tetracycline (Tet and Dox) or amphenicols (Chl and Ffc) (Fig. 4). In contrast, the addition of Tet and Dox resulted in 6.4- and 8.1-fold increases in the mRNA expression of *arfA*, respectively, whereas that of Chl and Ffc increased by 12.2- and 11.3-fold, respectively. Addition of Ery resulted in a 3.4-fold increase, while that of Str, Par, or Azm exerted no substantial effects on mRNA expression of *arfA*.

To confirm this, we performed qPCR experiments using MG1655 in the same manner. Similar results were obtained for MG1655. Specifically, *arfA* mRNA levels were increased by Chl and Ffc by approximately 24-fold (Supplementary Fig. 9).

These results revealed that mRNA expression of *arfA* is induced by tetracyclines or amphenicols that were observed to significantly impair growth of Δ*arfA*, thus suggesting an intrinsic role of ArfA in rescue of stalled ribosomes caused by tetracyclines and amphenicols. The addition of antibiotics also decreased the expression level of tmRNA by approximately half. This change may be helpful for increasing active ArfA as described in detail in the “Discussion” section.

## Discussion

**Fig. 5:**
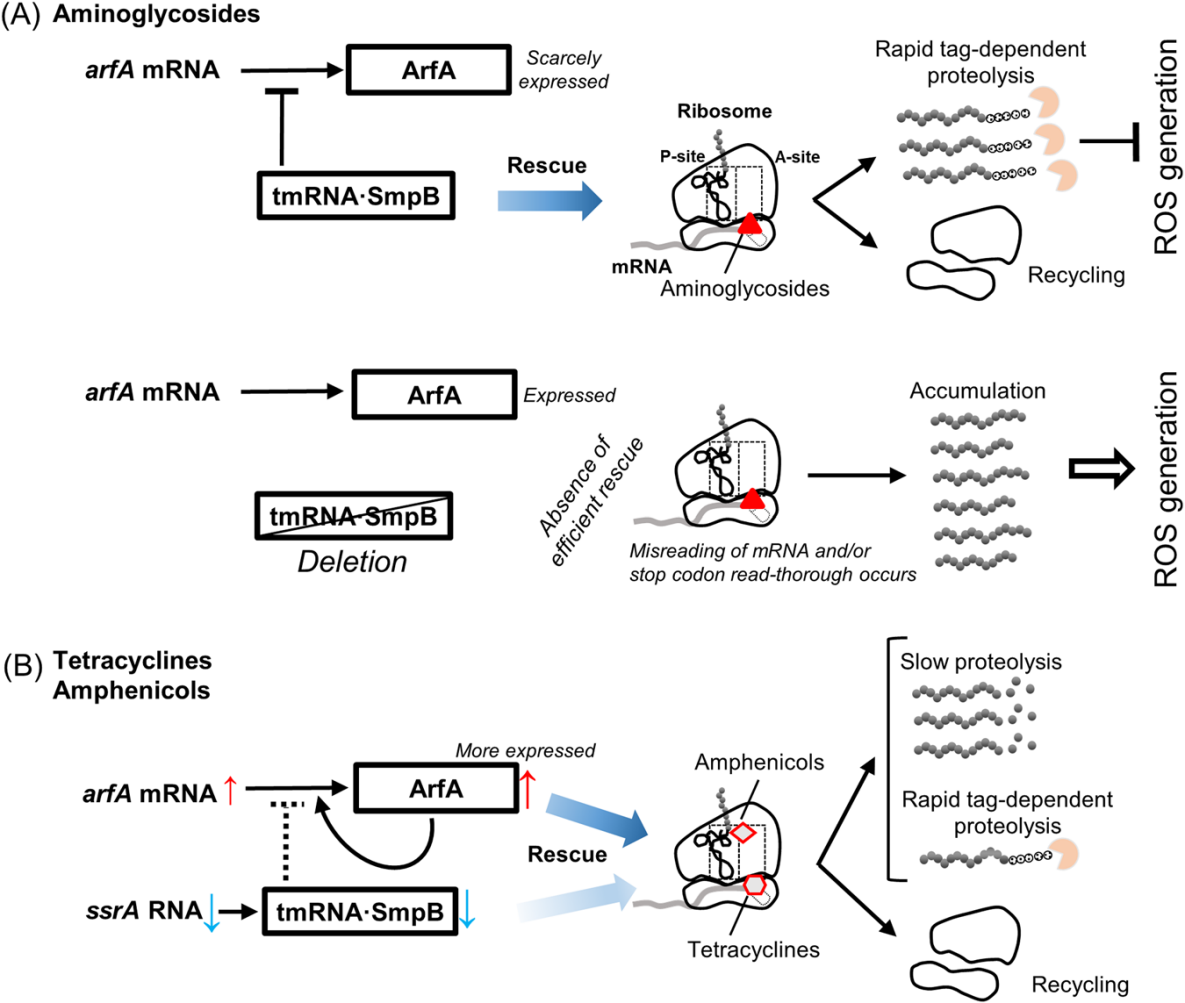
Schematic diagrams of roles of tmRNA·SmpB and ArfA in rescue of antibiotic-dependent stalled ribosomes in *E. coli.* **A** The stalled-ribosome rescue process in the presence of the aminoglycosides. Upper: tmRNA·SmpB rescues stalled ribosomes caused by the aminoglycosides by adding the tag peptide to the nascent polypeptides (indicated by a wide blue arrow). Tag-dependent proteolysis of the resultant aberrant proteins rapidly occurs so that ROS generation can be suppressed. Lower: in the absence of tmRNA·SmpB, ArfA is expressed; however, it does not help to rescue ribosomes exposed to aminoglycosides. Misreading of mRNA and/or stop codon read thorough occurs, and consequently, aberrant proteins accumulate in cells (see the text). This results in an increase in intracellular ROS levels that may lead to severe growth reduction or cell death. **B** The stalled-ribosome rescue process in the presence of tetracyclines and amphenicols. Red and blue vertical arrows indicate increase and decrease in mRNA induced by the antibiotics, respectively. A dotted T-shaped lines indicate a weakening of inhibition. When ArfA begins to increase in cells, this causes a positive feedback mechanisms where ArfA further increases itself (indicated by a rotated arrow). Although full-length ArfA protein that is produced when *arfA* mRNA is not cleaved by RNase III may also be more expressed, it is unstable and degraded immediately, as it possesses an extremely hydrophobic C-terminal tail that leads to aggregation and degradation of the full-length protein^23^. ArfA can rescue stalled ribosomes caused by the bacteriostatic antibiotics even more efficiently (a blue arrow) than can tmRNA·SmpB (a light blue arrow). Note that neither tetracyclines nor amphenicols induce ROS generation.

Our findings indicate that when the concentration of an aminoglycoside exceeds a certain level in the medium, intracellular ROS levels drastically increase, and accordingly, the tag-specific proteolysis mediated by tmRNA·SmpB becomes more essential for growth (Fig. 5A). A typical scenario explaining what occurs in bacterial cells under aminoglycoside stress is as follows^66,67^. Aminoglycosides bind to the decoding site of the 16S rRNA in the ribosomal small subunit and interfere with the selection of cognate tRNAs during translation. Consequently, the ribosome binding of each antibiotic induces misreading of mRNA and/or stop codon read-thorough. Subsequently, abnormal proteins into which incorrect amino acids are incorporated may be released from ribosomes if the mRNA escapes cleavage (as described in the “Introduction”). The accumulation of such mistranslated or misfolded proteins in cells is considered a starting point of several cell death pathways. Accordingly, certain aberrant proteins are inserted into the plasma membrane, leading to membrane damage and breakdown. This envelope stress causes excessive ROS generation via several signaling and reaction pathways. Excessive ROS levels damage biological macromolecules such as lipids, nucleic acids, and proteins, thus altering metabolism, respiration, and iron homeostasis, and this further increases ROS levels and continues a negative cycle. When this damage is too severe to repair, it disrupts cell integrity or causes death. Accordingly, it appears that above a certain antibiotic concentration, ROS suppression becomes more important for bacterial growth in the presence of aminoglycosides than stalled-ribosome rescue does. Δ*ssrA* cells that could not suppress ROS were more likely to experience a negative cycle. Concurrently, by binding directly to membranes, some (but not all) aminoglycosides at high concentrations increase membrane permeability or destroy the membrane, ultimately leading to cell death^68,69^. If expressions of any proteins related to membranes are regulated by tmRNA·SmpB^70^, aminoglycosides may exert a more destructive influence on membranes of Δ*ssrA* than those of the wild-type strain. Further studies are necessary to fully understand physiological processes in which tmRNA·SmpB acts in the presence of aminoglycosides that possess modes of action that are quite complex. It is interesting to note that two roles of tmRNA·SmpB, stalled ribosome rescue and tag-dependent proteolysis, correspond to two properties of aminoglycosides that include ribosome stalling and ROS generation. There may be more of an evolutionary relationship between tmRNA·SmpB and aminoglycosides than expected.

**Fig. 6:**
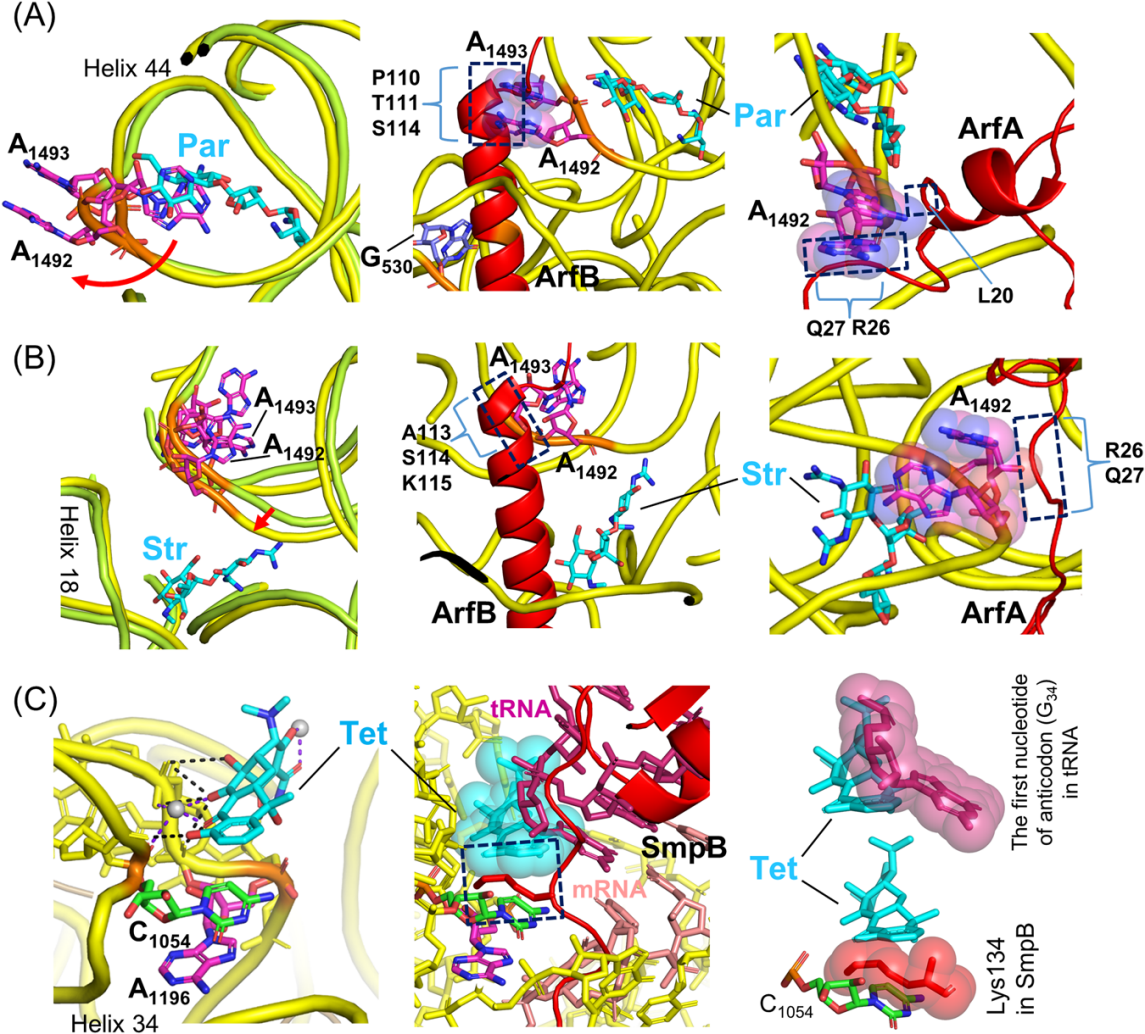
Possible models in which each antibiotic that binds to 16S rRNA inhibits the binding of specific ribosome rescue factors to the empty A site of a ribosome. **A** Left: Close-up views of superimposition of 16S rRNA of the small ribosomal subunit (PDB ID: 4V4Q) (green) and that bound with Par (4V5Y) (yellow). The backbone trace of 16S rRNA is depicted in either color; only A_1492_ and A_1493_ nucleotides in helix 44 are presented. A red arrow indicates changes in the base orientation caused by the Par binding. Middle and Right: Close-up views of superimposition of 16S rRNA bound with Par (4V5Y) and that with ArfB (6YSU) (middle) and that of 16S rRNA bound with Par and that with ArfA (5H5U) (right). van der Waals surfaces are presented for the bases of A_1492_ and A_1493_. The ribbon representations of ArfB and ArfA are depicted in red. Locations of putative clashes are surrounded by dotted skeleton boxes. Additionally, positions of putative clashed amino acid residues are indicated. All PDB data used in this Figure are from *E. coli* with the exception of 4DR3 (Str) from *Thermus thermophiles*. PyMOL (PyMOL Molecular Graphics System, Version 2.0, Schrödinger, LLC) was used to visualize and analyze all structures. **B** Left: Close-up views of superimposition of 16S rRNA (4V4Q) (green) and that bound with of Str (4DR3) (yellow). Only A_1492_ and A_1493_ nucleotides in the Str-bound 16S rRNA are indicated by black lines. A red arrow indicates a shift of the backbone of helix 44 laterally in the direction of ribosomal protein S12 and helix 18. Middle and Right: Close-up views of superimposition of 16S rRNA bound with Str (4DR3) and that with ArfB (6YSU) (middle) and that of 16S rRNA bound with Str and that with ArfA (5H5U) (right). **C** Left: Close-up views of the interaction between 16S rRNA and Tet (5J5B). Tet stacks with C_1054_ that is involved in decoding. The gray balls indicate Mg^2+^. The black and purple dashed lines indicate hydrogen bonds and electrical interactions, respectively. Middle: Close-up view of the superimposed small subunits structures focusing on the Tet-binding (5J5B), the SmpB-binding (7AC7), and the tRNA/mRNA binding (4V66). tRNA and mRNA are depicted in warmpink and salmon, respectively. A van der Waals surface is presented for Tet. Right, Top: Putative clash between Tet (5J5B) and the first nucleotide of anticodon (G_34_) in tRNA (4V66). Right, bottom: Putative clashes between Lys134 in SmpB (7AC7) and Tet (5J5B) and between Lys134 and C_1054_ in 16S rRNA (5J5B). van der Waals surfaces are presented for G_34_ and Lys134.

In contrast to the requirement for tmRNA·SmpB, the results suggest that neither ArfA nor ArfB can efficiently rescue aminoglycoside-dependent stalled ribosomes. Many aminoglycosides bind near the decoding region of the 16S rRNA helix 44, where conformational changes of specific nucleotides occur. Bound Par flips A_1492_ and A_1493_, two of the most important nucleotides involved in decoding, out of the helix structure^71^ (Fig. 6A, left). Bound Str shifts the decoding region of helix 44 laterally in the direction of ribosomal protein S12 and helix 18 instead of flipping them out^72^ (Fig. 6B, left). Comparison between the 16S rRNA structures bound with Par or Str and with ArfB or ArfA suggests that the filliped-out bases (Par) and the shifted backbone (Str) of 16S rRNA may overlap with the backbones of specific residues of the C-terminal regions of ArfB and ArfA, thus not allowing for accommodation of ArfB and ArfA into the A sites of stalled ribosomes (Fig. 6A, B, middle and right and Supplementary Fig. 10). However, further structural studies are required to confirm this hypothesis.

It was also observed that tmRNA·SmpB is not preferred over ArfA for ribosome rescue in the presence of tetracyclines or amphenicols (Fig. 5B). This may be explained by a common defect that the tRNA properties of tmRNA·SmpB and tRNAs share in the presence of these antibiotics. Tet primarily binds to the decoding region of 16S rRNA in the small ribosomal subunit. Bound Tet stacked with C_1054_, which is involved in decoding, collides with the anticodon loop of a fully accommodated aminoacyl-tRNA (Fig. 6C), ultimately pushing aminoacyl-tRNA out of the A site^73,74^. According to the structure of the accommodated *trans*-translation complex on the *E. coli* stalled ribosome^10^, bound Tet may crash with a few residues, particularly Lys134, of the C-terminal tail of SmpB binding to the mRNA entry channel, resulting in reduction of efficiencies of alany-tmRNA·SmpB to become stably accommodated in the A-site, as is the case with tRNAs (Fig. 6C and Supplementary Fig. 11). However, no Tet interaction blocks appeared in the C-terminal regions of ArfA or ArfB (Supplementary Fig. 11). Chl binds to the peptidyl transferase center in the large ribosomal subunit, where the aromatic ring of ribosome-bound Chl overlaps with the aminoacyl moiety of the incoming aminoacyl-tRNA^75–77^. This overlap inhibits peptidyl transfer and peptide bond formation. It is plausible that the interference of Chl with aminoacyl-tRNAs occurs with alanyl-tmRNA·SmpB. Amphenicols as well as tetracyclines are most likely to hinder the tRNA mode of the tmRNA·SmpB action on the stalled ribosome, consequently decreasing the efficiency of tmRNA·SmpB in ribosome rescue (note that macrolides do not hinder tRNA functioning in terms of their mechanism of action). Thus, ArfA may be considered a compensatory factor for the defect of tmRNA·SmpB that is revealed in the presence of the tetracyclines and amphenicols rather than simply as an alternative rescue factor to tmRNA·SmpB.

Furthermore, qPCR experiments demonstrated that tetracyclines (Tet and Dox) and amphenicols (Chl and Ffc) increased mRNA expression of *arfA* significantly. Interestingly, these antibiotics decreased the expression of *ssrA* to a certain extent. Considering the regulatory mechanism of ArfA expression, a decrease in the expression level of tmRNA may help increase the expression of active ArfA (Fig. 5B). Notably, the expression of plasmid-derived ArfA protein with an N-terminal His tag increases in cells as the amount of Chl added to the medium increases, although the ArfA protein is barely detected in the absence of Chl^23^. These findings suggest that ArfA is involved in the intrinsic protection against tetracyclines and amphenicols in *E. coli*.

The growth reduction of Δ*arfA* in the presence of tetracyclines and amphenicols could be complemented by overexpression of tmRNA·SmpB or ArfB. This result indicates that if the levels of tmRNA·SmpB, or ArfB are increased in cells, they can efficiently rescue Tet- or Chl-dependent stalled ribosomes. Of note, in *Mycobacterium smegmatis* and *Thermotoga maritima* that possess neither ArfA nor ArfB, tmRNA levels are considerably induced by ribosome-targeting antibiotics (Tet, Chl, Ery, Clr, and Str in *M. smegmatis*; Tet in *T. maritima*)^78,79^. In *S. coelicolor* that lacks *arfA*, *arfB* is significantly upregulated in the presence of Tet by the WblC/WhiB7 transcription factor that is required for intrinsic resistance to ribosome-targeting antibiotics^80,81^. Regardless of the type of bacteria, the total rescue system for stalled ribosomes may be programmed to compensate for the tmRNA·SmpB weakness through certain mechanisms such as increasing the quantity of ribosome rescue factors including tmRNA·SmpB. Interestingly, *Bacillus subtilis* and *Francisella tularensis* possess different RF-dependent rescue factors (BrfA and ArfT, respectively)^82,83^. As BrfA, ArfT, and ArfA exhibit little sequence homology among one another, they appear to have evolved convergently. It would be intriguing to examine whether BrfA and ArfT play a role similar to that of ArfA in the presence of ribosomes stalled by specific antibiotics.

This study revealed that tmRNA·SmpB and ArfA play different roles in rescuing antibiotic-dependent stalled ribosomes depending on the type of ribosome-targeting antibiotic. Thus, they do not possess an alternative relationship in terms of rescuing such stalled ribosomes. Consequently, the combination of the two ribosome rescue factors presumably confers *E. coli* with the ability to efficiently rescue diverse types of antibiotic-dependent stalled ribosomes, directly increasing the probability of its survival in nature. This study also highlights that tmRNA·SmpB-mediated tag-specific proteolysis is vital for growth in the presence of aminoglycosides involved in ROS generation. Probably, tmRNA·SmpB plays a crucial role in decreasing the background ROS levels in cells under specific conditions of ROS generation, which are induced by any cause and not just by antibiotics. Further studies are required to understand the relationship between ribosome rescue factors and various types of stress-induced translation inhibition in different bacteria, which may explain why the combination of ribosome rescue factors differs depending on the bacterial species. A better understanding of the ribosome rescue system in individual bacteria may help to assess the characteristics of bacteria-specific antibiotic resistance to improve antibiotic efficacy.

## Methods

### Chemicals

Antibiotics were purchased from Fujifilm Wako (kanamycin sulfate, gentamicin sulfate, streptomycin sulfate, and tetracycline hydrochloride), Sigma-Aldrich (paromomycin sulfate and chloramphenicol), Tokyo Chemical Industry (azithromycin dihydrate, erythromycin, clarithromycin, oxytetracycline hydrochloride, doxycycline hyclate, thiamphenicol, and florfenicol), and Nacalai Tesque (ampicillin sodium salt).

### Strains, plasmids, and medium

*E. coli* strains and plasmids used in this study are listed in Supplementary Tables 2 and 3, respectively. For the culture medium, L-Broth powder (MP-Biomedical) and NaCl were mixed such that the resultant LB medium contained 0.5% (w/v) bacto-yeast extract, 1% (w/v) bacto-tryptone, and 1% (w/v) NaCl. The medium was finally adjusted to pH 7.0 with 1 N NaOH.

### Construction of gene deletions in *E. coli* SE15

Each gene deletion mutant from SE15 was constructed using a suicide vector-mediated genome editing system according to a previous report^45^. The pABB-CRS2 plasmid was used as a mobilizable suicide vector and included the key features described in Supplementary Fig. 12A. The vector can only replicate in specific strains that express *pir*-encoded π protein (SM10λ *pir* in this study), as this protein is required for replication via the R6K γ origin. Additionally, the vector carried *sacB* that encodes levan sucrase and confers sensitivity to sucrose, and it also possessed the mob site from the RP4 plasmid for transconjugation. Briefly, the pABB-CRS2 vector was linearized with NotI and NcoI. The upstream and downstream regions (~1000 bp) of the target gene were amplified by PCR using F1 and F2 primers (for example, Del-ssrA(F1)-F/-R and Del-ssrA(F2)-F/-R) and genomic DNA as the template (Supplementary Fig. 12B). Two PCR primers for target gene deletion were designed such that the majority of the ORF was removed to create a short ORF (Supplementary Fig. 13). Accordingly, each PCR product possessed 15-bp extensions at the 5ʹ end that were complementary to the ends of the linearized vector and 20-bp extensions at 3ʹ end that were complementary between each primer sequence. The two resulting PCR products were cloned into the linearized vector using an In-Fusion cloning kit (Takara). This resultant vector was transformed into donor strain *E. coli* SM10λ *pir*. Donor cells harboring the plasmid were mixed with acceptor strain SE15 cells, and plasmids from the donor cells were trans-conjugated into the acceptor cells. Site-specific recombination (the first crossing over) occurred via Campbell-type integration so that the vector was integrated into the genome of the acceptor cells. The mixture was cultivated in LB liquid medium without Amp for approximately 3 h and then spread onto M9 minimal medium agar plates containing Amp. The following day, for accuracy Amp^R^ positive clones were assembled, and the previous procedure was repeated. The next day, several colonies were individually inoculated into a special liquid LB medium containing 5% sucrose but not NaCl. The cultures were cultivated for 10 ~ 16 h, appropriately diluted, plated on agar plates created using special LB medium, and incubated overnight. The second crossover occurred when the vector was removed from the genome, and this was driven by the *sacB* selection marker. As cells harboring the vector could not survive in the presence of sucrose, the colonies on the plate possessed the wild-type or mutant genotype. Among the sucrose-resistant colonies, the mutant of interest was selected using PCR. The primer sequences are listed in Supplementary Table 4.

### Construction of plasmids for expression of ribosome rescue factors

A plasmid expressing active ArfA composed of 54 amino acid residues was constructed as follows. A 395-bp DNA fragment containing a truncated *arfA* ORF and a 200-bp region upstream of the ORF were amplified by PCR (KOD-Plus-Neo DNA polymerase [TOYOBO]) using SE15 genomic DNA as a template and two primers that included ArfA162-F and ArfA162-R. A linearized pBR322 vector was generated using the inverse PCR method by two primers that possessed EcoRI and SalI sites at the 5′ and 3′ ends, respectively. The DNA fragment was cloned into the linearized pBR322 vector using the In-Fusion cloning kit, and consequently, the majority of the tetracycline resistance genes and the promoter were replaced with the DNA fragment. For pArfB and pSmpB, the *arfA* ORF in pArfA was replaced with *arfB* and *smpB* ORFs, respectively, using inverse PCR and in-fusion cloning. Regarding pSsrA/SmpB, the DNA fragment from the 193 bp region upstream of the *smpB* ORF to the 131-bp region downstream of the *ssrA* ORF was amplified by PCR with two primers (SsrA/SmpB-F and SsrA/SmpB-R) and cloned into the linearized pBR322 vector in the same manner as mentioned above.

The plasmid expressing tmRNA pMW-SsrA was constructed as follows: pMW118 was selected as the expression vector, as it has often been used as an expression vector for *ssrA* in previous studies (see, e.g., papers^84,85^). A major difference between the two plasmids was the copy number (~5 for pMW118 vs. ~20 for pBR322). A 559-bp DNA fragment containing the *ssrA* region, the 5ʹ flanking region (60 bp), and the 3′ flanking region (100 bp) was amplified by PCR using the MG1655 genomic DNA as a template with two primers that included SsrA-F and SsrA-R (the *ssrA* sequence are identical between MG1655 and SE15). The DNA fragment was cloned into the linearized pMW118 vector that possessed HindIII and SphI sites at the 5′ and 3′ ends in the same manner as mentioned above. For pMW-SsrA^DD^, site-directed mutagenesis was used to alter GCAGCT (corresponding to Ala-Ala) to GATGAT (corresponding to Asp-Asp) in the ORF of *ssrA* using two primers (SsrA^DD^-F and SsrA^DD^-R).

### Growth measurements and IC_50_ determinations

The wild-type and mutant strains were precultured in 5 ml of liquid LB medium in the absence of any antibiotics at 34 °C on a reciprocal shaker (25 mm shaking diameter at 130 strokes/min) for 16 h. Each culture was inoculated into 25 ml of LB medium in CELLSTAR CELLreactor 50 ml tubes (Greiner) that possess a filter screw cap for optimal ventilation of the tubes with the starting optical density at 600 nm (OD_600_) of 0.001. Simultaneously, each antibiotic was added to the medium at the indicated concentration. The strains were aerobically grown at 37 °C on a reciprocal shaker with the tube fixed at an angle of approximately 30°. Cell growth was assessed by measuring the OD_600_ values at a specific time depending on the individual experiments (see each legend) using a spectrophotometer (U-1500, Hitachi). For plasmid-containing strains, ampicillin was added to the LB medium for plasmid retention of pBR322 or pMW118. It should be noted that whether the preculture temperature is 34 °C or 37 °C, a series of results are essentially the same.

The half-inhibitory concentration (IC_50_) of each antibiotic was determined according to the following dose-response curve (Hill equation): *Y* (%) = 100 / (1 + (log_10_ IC_50_ / log_10_ *X*)^*h*^), where *h* is the Hill coefficient, *X* is the antibiotic concentration, and *Y* (%) is the ratio of the OD_600_ value measured in the presence of each concentration of antibiotic to that in the absence of any antibiotic. Curve fitting was performed by nonlinear regression using GraphPad Prism 9.3.1 for Windows (GraphPad Software).

### Detection of viability of antibiotic-treated cells

Bacterial viability was assessed using a bacterial viability detection kit that included two fluorescent probes (CTC and DAPI) (Dojindo). According to the procedure described in the above paragraph, the strains were cultured for 5 h in the absence or presence of each antibiotic at the IC_50_ concentrations. After centrifugation and washing twice with PBS, each pellet was resuspended in 1 ml of PBS to an OD_600_ of 1.0. The CTC/DAPI double staining was performed according to the manufacturer’s protocol. Fluorescence images of the samples were captured using a fluorescence microscope (BZ-9000, Keyence) with excitation and emission filters for CTC at 560/40 and 630/60 nm and those for DAPI at 360/40 and 460/60 nm, respectively. Cell counting based on the images was performed automatically using ImageJ2/Fiji software (ver. 2.14.0)^62^. The Yen thresholding algorithm was applied to the fluorescence images^86^.

### Antibiotic susceptibility testing

For antibiotic susceptibility assessments, MICs were determined using the broth microdilution method according to a previous protocol^51^. Briefly, *E. coli* strains were first streaked onto LB agar plates, and single colonies were picked and incubated overnight in liquid LB medium. The media was diluted with fresh LB media to 1 × 10^7^ colony forming unit (CFU)/ml, and an OD_600_ of 1.0 was determined to correspond to 7 (±1) × 10^8^ CFU/ml in triplicate in this study. The bacterial suspension of 100 µl was added to each well of a 96-well microplate (round bottom, AS ONE) with 100 µl of a series of 2-fold dilutions of an antibiotic. The resulting concentration of each strain was 5 × 10^6^ CFU/well. The microplates were incubated at 37 °C for 16 h without shaking. The lowest concentration of antimicrobials that inhibited the visible growth of each strain was identified as the MIC value.

### Detection of intracellular ROS and cell counting of ROS-positive cells

Intracellular ROS levels were assessed using the ROS Assay Kit that included a fluorescent probe and a photooxidation-resistant derivative of DCFH-DA (Dojindo). The wild-type and three ribosome rescue factor-deficient strains were grown for 2.5 h in the absence of antibiotics according to the procedure described above. Then, Str, Par, or Tet was added to the medium at 1.5-fold the IC_50_ concentrations, and the cultures were incubated for 3 h. The cells were harvested by centrifugation and washed twice with phosphate-buffered saline (PBS). The pellets were resuspended in 100 µl of the ROS Assay working solution containing the fluorescent probe, and the suspensions were incubated for 30 min at 37 °C. After centrifugation and washing twice with PBS, each pellet was resuspended in PBS to an OD_600_ of 2.0. Bright-field and fluorescence images of the samples were captured using the fluorescence microscope with excitation and emission filters of 470/40 nm and 535/50 nm, respectively. Cell counting based on the images was performed automatically using ImageJ2/Fiji (ver. 2.14.0)^62^. The Yen and Otsu thresholding algorithms were applied to bright-field and fluorescence images, respectively^86,87^. In Fig. 3D, the data were fitted to the following equation of the variable slope sigmoidal dose-response curves: *Y* = Bottom + (Top – Bottom) / (1 + (EC_50_ / *X*)^*h*^), where *h* is the Hill coefficient, EC_50_ is the midpoint of the curve, *X* is the antibiotic concentration, *Y* is the percentage of fluorescence-positive cells, and Top and Bottom are the plateaus in the units of the *Y*-axis. Curve fitting was performed using nonlinear regression in GraphPad Prism 9.3.1.

### Quantitative reverse-transcription PCR

The strains were grown according to the procedure described above. When the culture medium reached an OD_600_ of 0.3 ~ 0.4, each antibiotic at double the IC_50_ concentration was added to the medium. Subsequently, the culture was continued for 30 min. Two milliliters of the medium were centrifuged at 5000 × *g* for 10 min, and the precipitated cells were washed. RNA was extracted from the cells of each strain using the TRIzol reagent according to the standard protocol. For total RNA, 5 ng of total RNA was used as template. qPCR was performed using a One-Step TB Green PrimeScript RT-PCR Kit II (Takara) on a LightCycler 480 System II (Roche Applied Science). *idnT* was used as an internal reference gene (see text). The qPCR results including threshold cycle (Ct) values were analyzed using LightCycler 480 system software based on the ΔΔCt method. The primer sequences used for qPCR are listed in Supplementary Table 5.

### Statistics and reproducibility

Specific information regarding the number of replicates and statistical analyses is included in each Figure legend.

## Supplementary information

Supplementary Information

## Data Availability

The datasets generated and/or analyzed in the current study are available from the corresponding author upon reasonable request.
