# Supplementary material for: Stalled ribosome rescue factors exert different roles depending on types of antibiotics in Escherichia coli
Source: NPJ Antimicrob Resist. 2024 Sep 2;2:22. doi: 10.1038/s44259-024-00039-2 (PMC11721466; doi:10.1038/s44259-024-00039-2)
Supplement: Supplementary file 1 — Supplementary Information [file 44259_2024_39_MOESM1_ESM.pdf]

Supplementary Information

Stalled ribosome rescue factors exert different roles depending on types of antibiotics in *Escherichia coli*

Mayu Mikami, Hidehiko Shimizu, Norika Iwama, Mihono Yajima, Kanako Kuwasako, Yoshitoshi Ogura, Hyouta Himeno, Daisuke Kurita and Nobukazu Nameki

This file contains Supplementary Figures 1-13 and Tables 1-5.

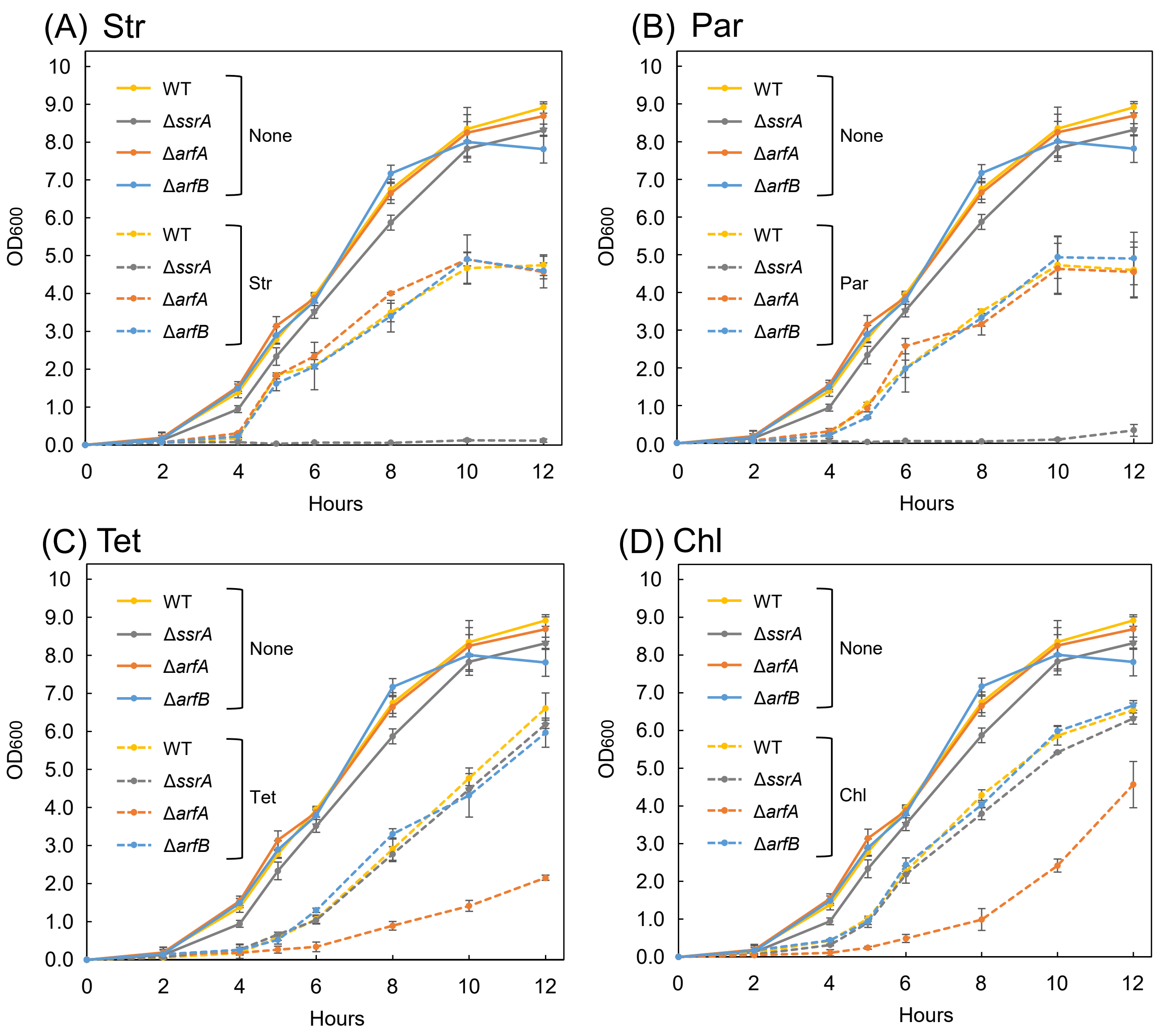

Supplementary Fig. 1. Time dependent growth curves of the wild-type and the three ribosome rescue factor-deficient strains with or without Str (A), Par (B), Tet (C), or Chl (D) at each IC_{50} concentration.

The sample preparation is described in the legend of Fig. 1. Data are presented as the mean ± standard deviation of three independent experiments.

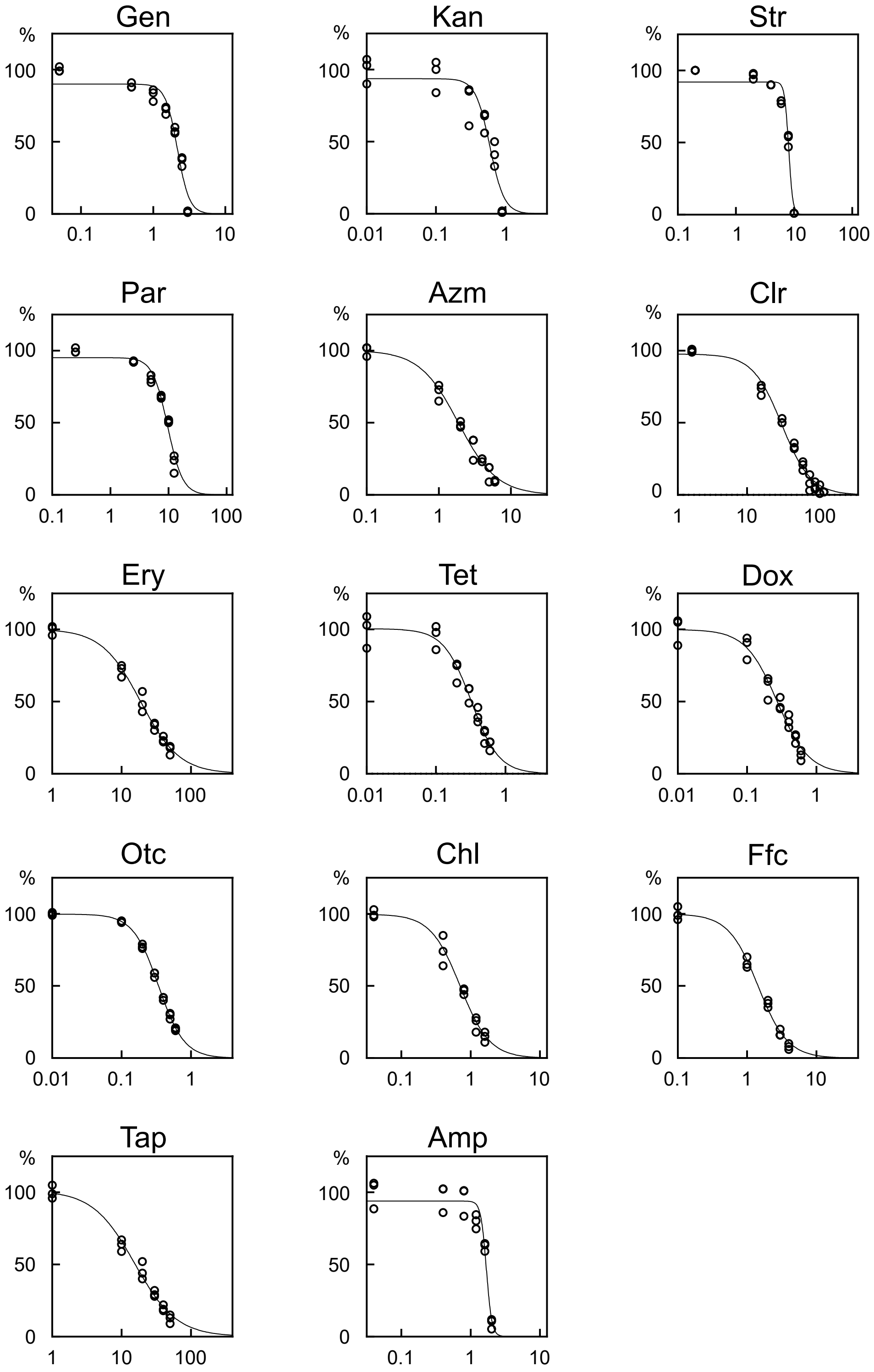

Supplementary Fig. 2. Antibiotic dose response curves for the wild-type SE15 strain. The wild-type strain was initially exposed to each antibiotic at the indicated concentrations at an initial OD_{600} of 0.001. Growth was assessed by measuring OD_{600} of the medium incubated at 37 °C for 8 h. The relative growth (%) was determined as the ratio of the OD_{600} value measured in the presence of an antibiotic at each concentration to that measured in the absence of antibiotics. The dose-response curves were fitted with the Hill equation using GraphPad Prism 9.3.1 as described in detail in the Methods section. Data are plotted from three independent experiments.

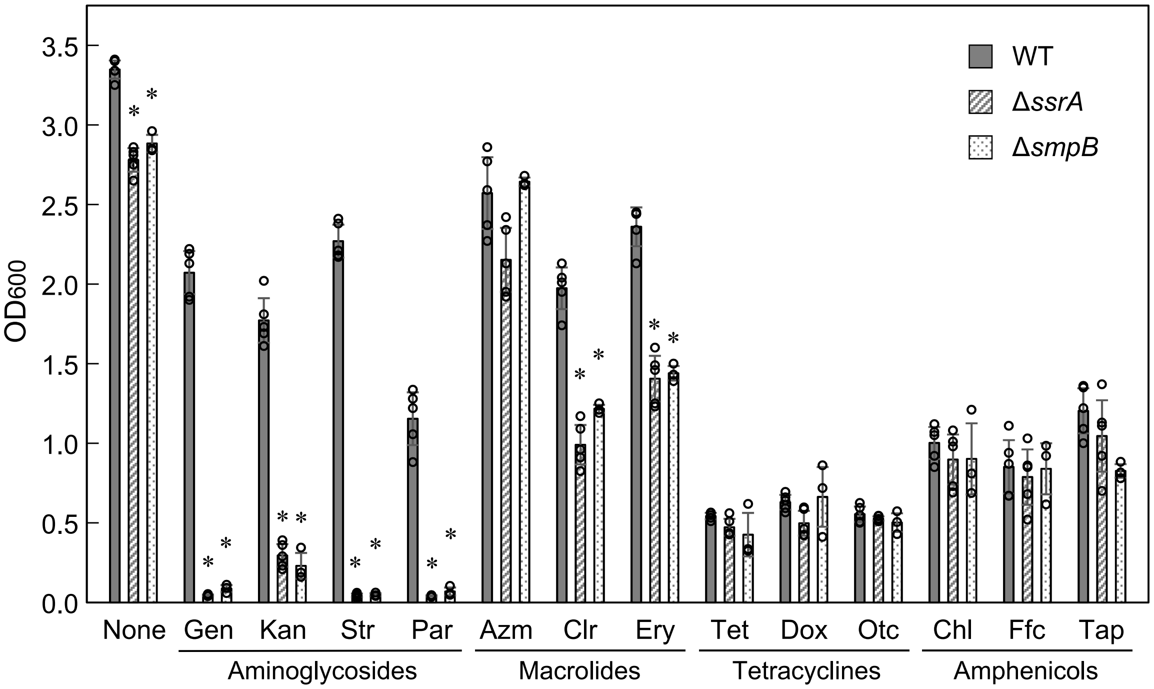

Supplementary Fig. 3. Comparison of inhibitory effects of ribosome-targeting antibiotics on growth among the wild-type, Δ*ssrA*, and Δ*smpB* strains.

The legend is the same as that for Fig. 1A. Data on Δ*smpB* are presented as the mean ± standard deviation of three independent experiments. Asterisks indicate significant differences compared to wild-type (Student's *t* test, $*P < 0.001$).

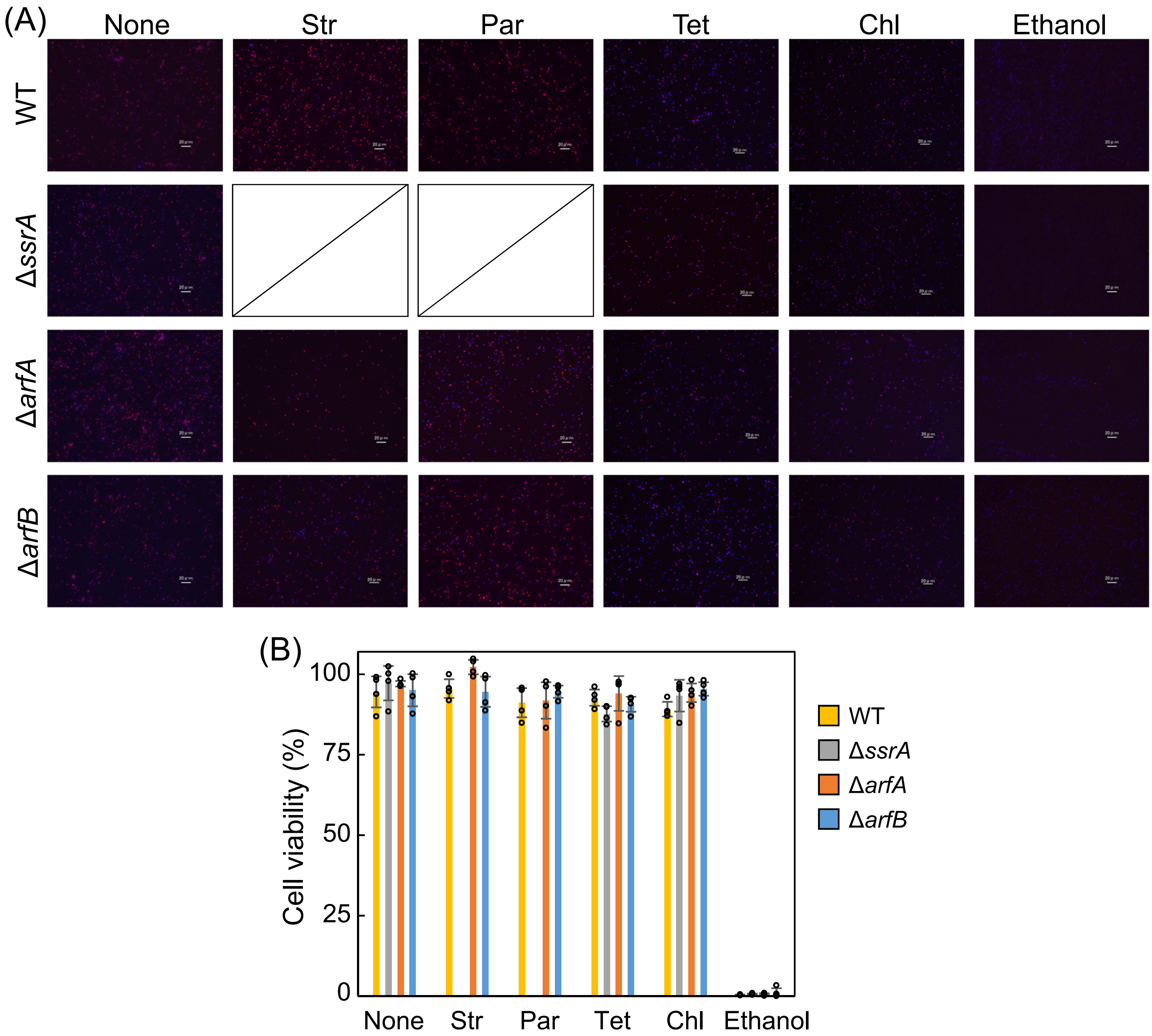

Supplementary Fig. 4. Cell viability of antibiotic-treated wild-type, Δ*ssrA*, Δ*arfA*, and Δ*arfB* strains.

(A) Representative CTC/DAPI double-staining images of the wild-type, Δ*ssrA*, Δ*arfA*, and Δ*arfB* cells that were cultured for 5 h in the presence of the indicted antibiotic at each IC_{50} concentration. Bacterial cells that possess respiratory activity are stained by CTC (red), whereas all bacterial cells are stained by DAPI (blue) regardless of whether they have a respiratory activity or not. Ethanol (50%) was used a negative control. The fluorescence images were obtained using a fluorescence microscope (magnification, ×40; scale bar: 20 μm). It should be noted that the absence of images for Δ*ssrA* in the presence of Str or Par is due to the observation that Δ*ssrA* could hardly grow in its presence at the IC_{50} concentration.

(B) Percentage of live cells of antibiotic-treated wild-type and Δ*ssrA*, Δ*arfA*, and Δ*arfB* cells. Two fields per sample were analyzed, and each time, at least 100 cells were counted. Two independent experiments were performed for the wild-type and the mutant strains (n = 4). The percentage of live cells was calculated as 100 × (the number of CTC-stained cells / that of DAPI-stained cells). Data are presented as the mean ± standard deviation of the four experiments.

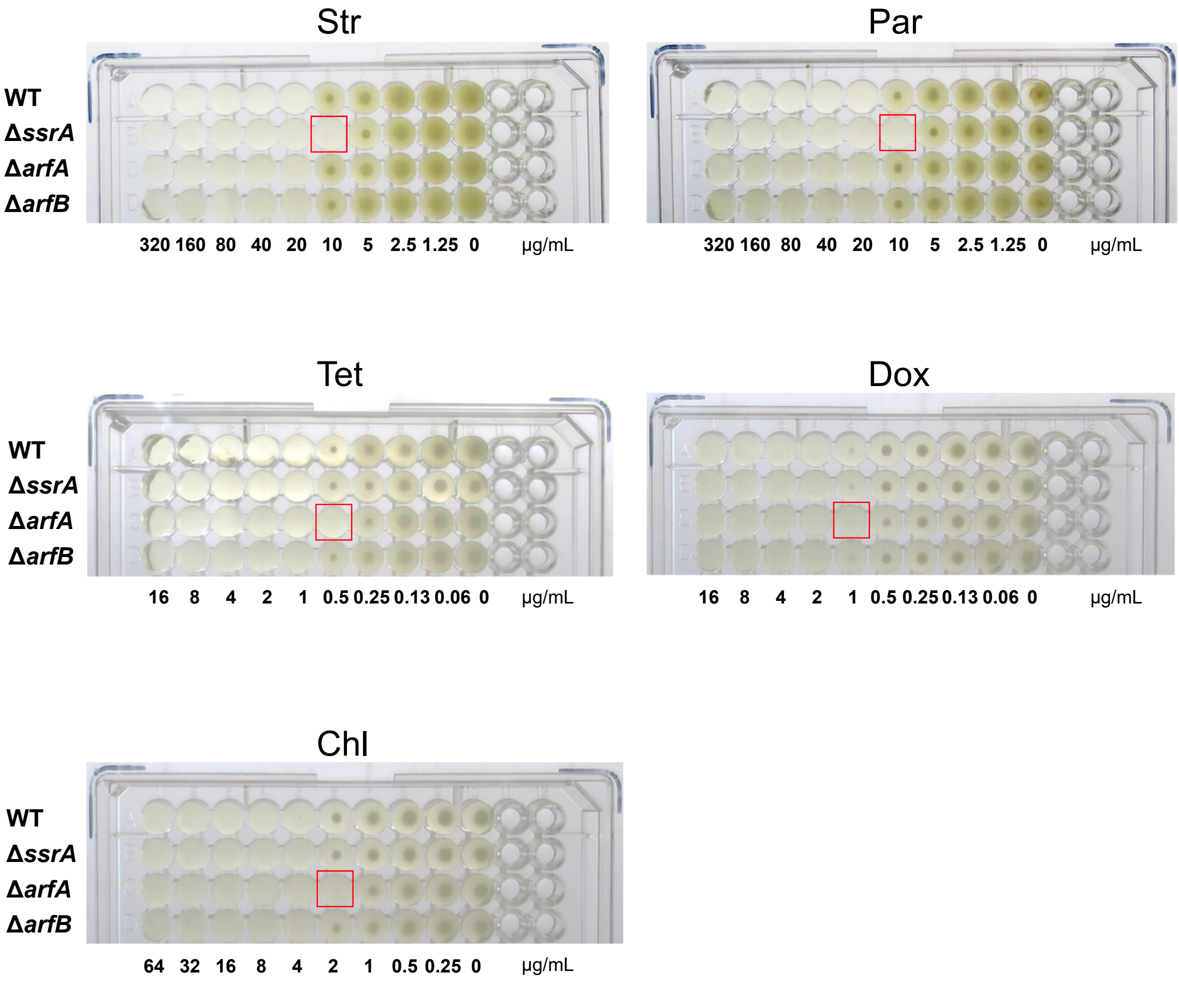

Supplementary Fig. 5. Representative microplates demonstrating the microbroth dilution approach using LB medium for determination of MICs of the five antibiotics against the wild-type, Δ*ssrA*, Δ*arfA*, and Δ*arfB* strains.

The concentration of each antibiotic per well is indicated below, and each dilution was 2 times more diluted than the previous dilution. Red boxes in the plates indicate MIC values that inhibited the visible growth of the strains that were most sensitive to each antibiotic. These experiments were repeated in triplicate.

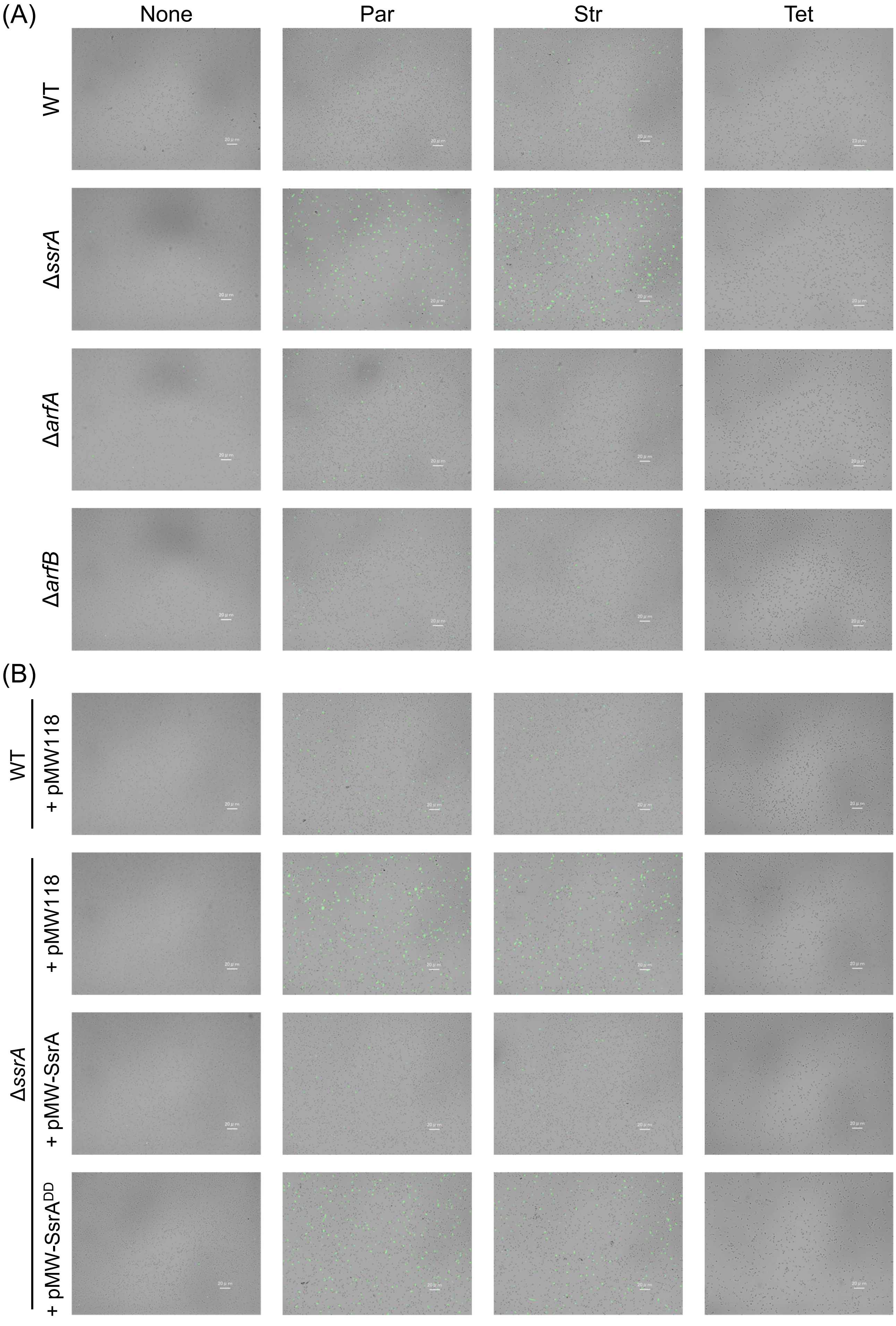

Supplementary Fig. 6 (legend on next page)

Supplementary Fig. 6. Representative merged bright-field and fluorescent microscopy images of antibiotic-treated cells stained with a photo-oxidation-resistant derivative of DCFH-DA.

(A) Representative images of antibiotic-treated wild-type, Δ*ssrA*, Δ*arfA*, and Δ*arfB* cells stained with the oxidant-sensing probe. Antibiotics are indicted at the top. The fluorescence images were obtained using a fluorescence microscope (magnification, ×40; scale bar: 20 μm). Data analysed using the images are shown in Fig. 3A.

(B) Representative images of the wild-type and Δ*ssrA* strains transformed with an empty plasmid, pMW-SsrA, or pMW-SsrA^{DD} and stained with the oxidant-sensing probe. Images regarding untreated and Par-treated wild-type and Δ*ssrA* transformant cells are identical to those in Fig. 3B. Data analysed using the images are shown in Fig. 3C.

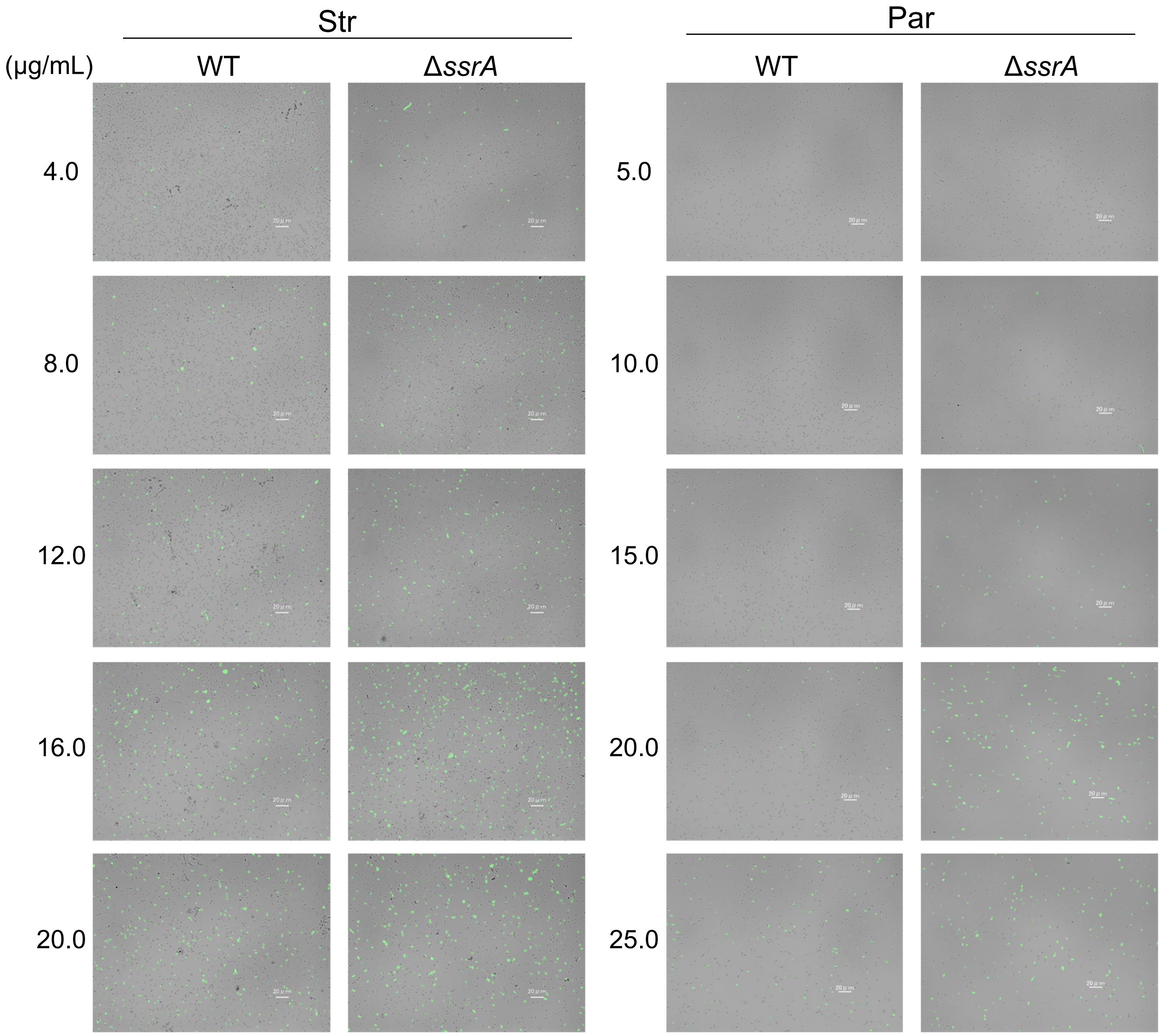

Supplementary Fig. 7. Representative merged bright-field and fluorescent microscopy images of Str- and Par-treated wild-type and Δ*ssrA* cells stained with DCFH-DA, depending on the antibiotic concentration.

Concentrations of each antibiotic used are presented. The fluorescence images were obtained using a fluorescence microscope (magnification, ×40; scale bar: 20 μm). Data analyzed using these images are provided in Fig. 3D.

(A)

	Ct-value									
	idnT		*smpB*		*ssrA*		*arfA*		*arfB*	
	Avg.	S. D.	Avg.	S. D.	Avg.	S. D.	Avg.	S. D.	Avg.	S. D.
None	25.03	0.43	21.68	0.62	10.91	0.22	20.31	0.55	22.40	0.60
Str	24.69	0.18	21.27	0.32	11.11	0.45	20.56	0.25	22.00	0.09
Par	24.87	0.21	21.40	0.22	11.09	0.32	19.85	0.84	21.96	0.39
Azm	25.19	0.37	21.64	0.34	11.38	0.36	20.72	0.55	22.07	0.76
Ery	25.16	0.09	23.76	0.29	12.33	0.15	19.45	0.28	23.97	0.18
Tet	25.37	0.17	22.13	0.33	11.59	0.49	17.92	0.24	23.52	0.35
Dox	24.84	0.18	21.45	0.42	11.57	0.24	17.35	0.37	22.46	0.29
Chl	25.20	0.28	21.69	0.64	11.57	0.20	16.90	0.54	23.40	0.18
Ffc	24.99	0.21	21.73	0.26	11.79	0.22	16.62	0.70	22.77	0.22

(B)

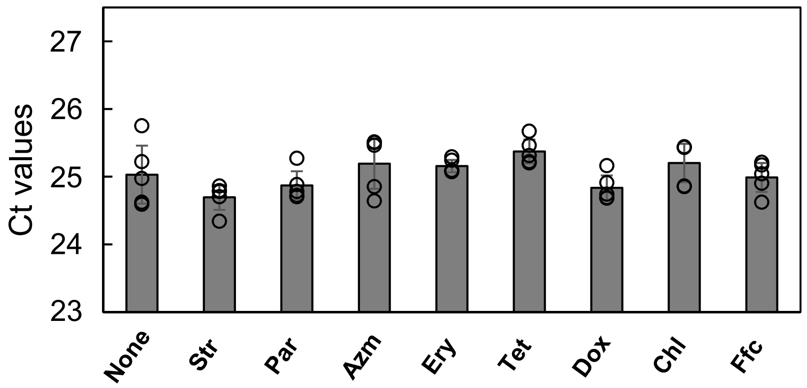

None vs.	Str	Par	Azm	Ery	Tet	Dox	Chl	Ffc
P-value	0.19	0.52	0.58	0.58	0.18	0.43	0.52	0.87

Supplementary Fig. 8. Ct values of the reference gene *idnT*, *smpB*, *ssrA*, *arfA*, and *arfB* in qPCR experiments using total RNA extracted from SE15 grown in the absence and presence of each antibiotic.

(A) The Ct values used for the calculations are summarized in the table. Data are presented as the mean ± standard deviation of five independent experiments.

(B) The Ct values for *idnT* are summarized in the graph (upper), and P values are presented in the table (lower). There were no statistically significant differences between the absence and presence of each antibiotic (Student's *t* test, $P > 0.1$).

(A)

	Ct-value									
	idnT		*smpB*		*ssrA*		*arfA*		*arfB*	
	Avg.	S. D.	Avg.	S. D.	Avg.	S. D.	Avg.	S. D.	Avg.	S. D.
None	24.94	0.19	22.71	0.53	11.13	0.07	20.66	0.32	22.78	0.02
Str	25.10	0.15	22.87	0.39	10.87	0.04	20.81	0.71	22.59	0.19
Par	25.06	0.15	23.14	0.43	11.11	0.36	20.20	0.34	22.91	0.08
Tet	24.86	0.08	23.15	0.64	11.73	0.19	18.08	0.05	23.56	0.22
Dox	24.94	0.09	22.71	0.41	11.97	0.18	17.49	0.25	23.59	0.22
Chl	24.80	0.19	23.39	0.59	12.36	0.35	15.24	0.63	24.28	0.41
Ffc	24.97	0.08	22.83	0.62	11.84	0.30	16.25	0.10	23.84	0.29

None vs.	Str	Par	Tet	Dox	Chl	Ffc
P-value	0.37	0.51	0.63	0.70	0.51	0.84

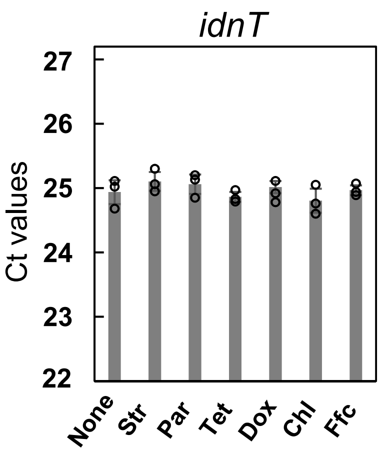

(B)

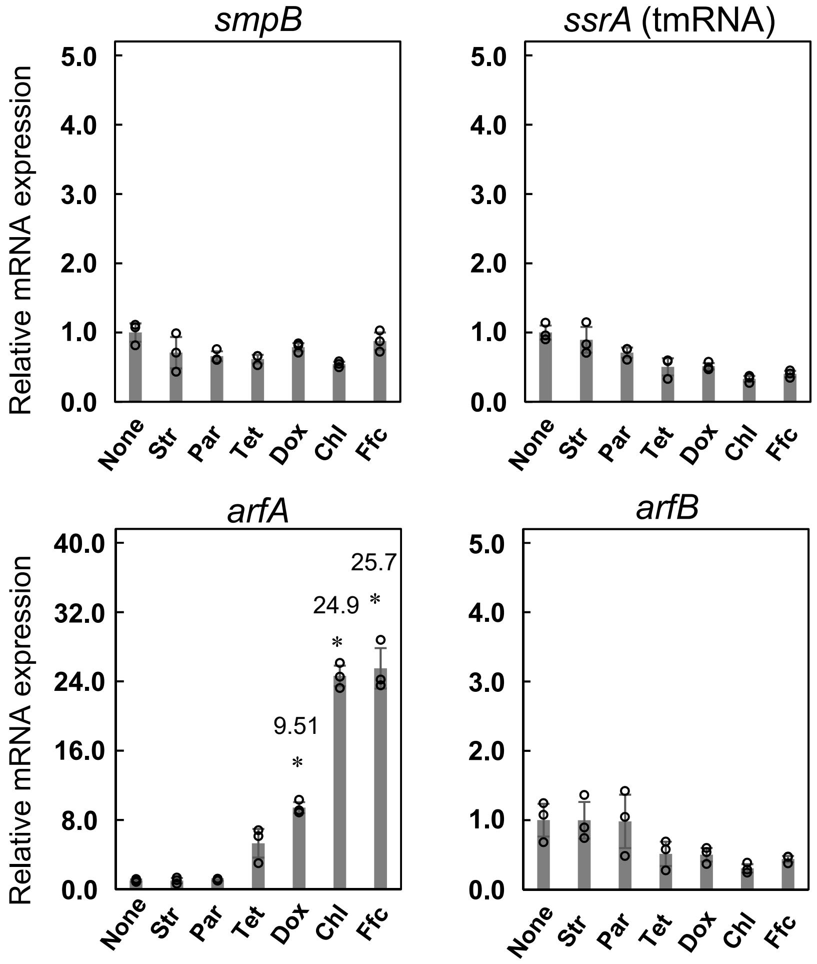

Supplementary Fig. 9. Effects of antibiotics on mRNA expression of *smpB*, *ssrA*, *arfA*, and *arfB* from total RNA extracted from MG1655 grown in the absence and presence of each antibiotic.

(A) The Ct values for the four genes and the reference gene *idnT* used for the calculations are summarized in the table (left, upper). Data are presented as the mean ± standard deviation of three independent experiments. The Ct values for *idnT* are summarized in the graph (right), and P values are presented in the table (left, lower). There were no statistically significant differences between the absence and presence of each antibiotic (Student's *t* test, $P > 0.1$). (B) Relative mRNA expression levels of *smpB*, *ssrA*, *arfA*, and *arfB* from MG1655. The experimental procedure and the calculation method were the same as those used for SE15. Data are presented as the mean ± standard deviation of three independent experiments. Asterisks indicate significant differences compared to wild-type (Student's *t* test, $*P < 0.001$).

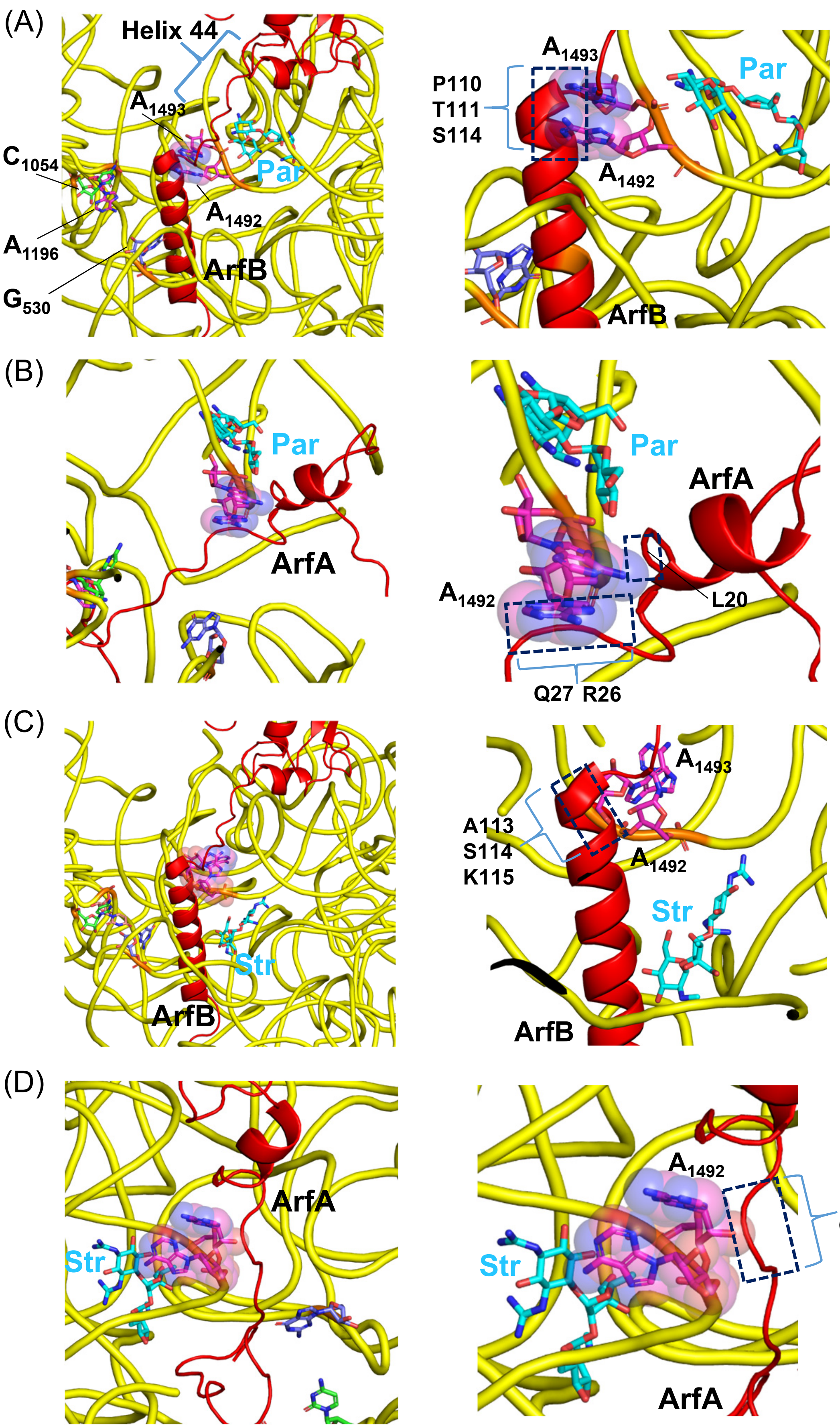

Supplementary Fig. 10. Possible clashes between Par/Str and ArfA/ArfB.
All figures in the right panels are identical to those in the middle and right panels of Fig. 6A and B (PDB ID: 4V5Y, 4DR3, 6YSU, 5H5U). The left panels provide enlarged views of the figures in the right panels so that the clash positions can be observed in the entire 16S rRNA.

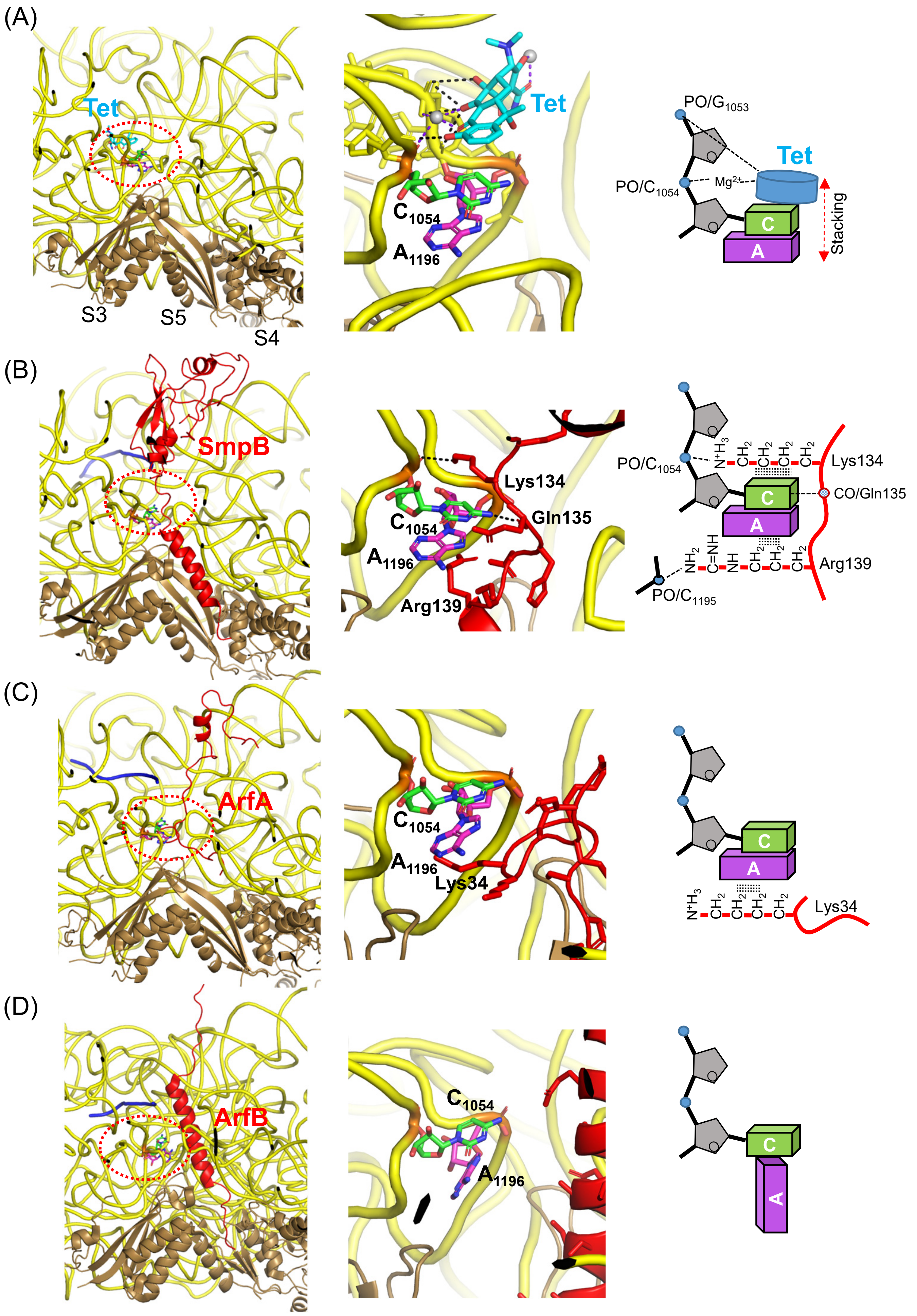

Supplementary Fig. 11 (legend on next page)

Supplementary Fig. 11. Comparison of the ribosome-binding modes among Tet, SmpB, ArfA, and ArfB.

Close-up view of the interactions of Tet (A), SmpB (B), ArfA (C), and ArfB (D) with *E. coli* ribosomes (PDB ID:5J5B, 7AC7, 5H5U, and 6YSU), respectively. *Left*: The backbone trace of 16S rRNA is depicted in yellow, whereas the ribbon representations of ribosomal proteins are depicted in sand. *Middle*: Close-up view of the interaction in the vicinity of C_{1054} and A_{1196} in the 16S rRNA that corresponds to the location circled by the red dashed line in each left panel. The figure in the middle panel in (A) is identical to that in the left panel in Fig. 6C. The gray balls indicate Mg^{2+}. The black and purple dashed lines indicate hydrogen bonds and electrical interactions, respectively. *Right*: Schematic of the interaction in the vicinity of C_{1054} and A_{1196} presented in the middle panel.

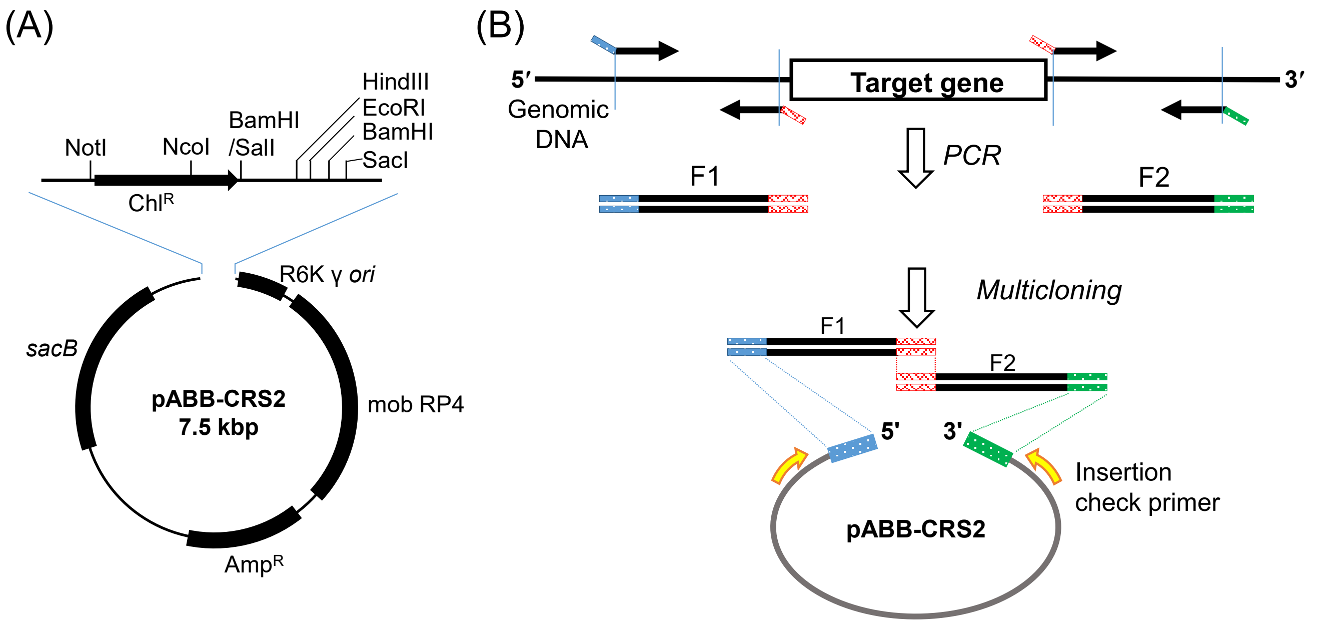

Supplementary Fig. 12. Diagram of a suicide vector-mediated genome editing system for gene deletion.

(A) Schematic representations of the suicide plasmid pABB-CRS2.

(B) Scheme for plasmid construction for gene deletion. Arrows indicate primer locations for PCR. The blue and green regions (15 nt) of the PCR products F1 and F2 (~ 1,000 nt) are homologous to the 5′ and 3′ end regions of the linear plasmid that is cut with restriction enzymes, respectively. The red regions of F1 and F2 (~ 20 nt) correspond to the region with which the target gene is replaced. The sequences of primers used, including the universal primers for insertion check (indicated as a yellow arrow), are presented in Supplementary Table 4.

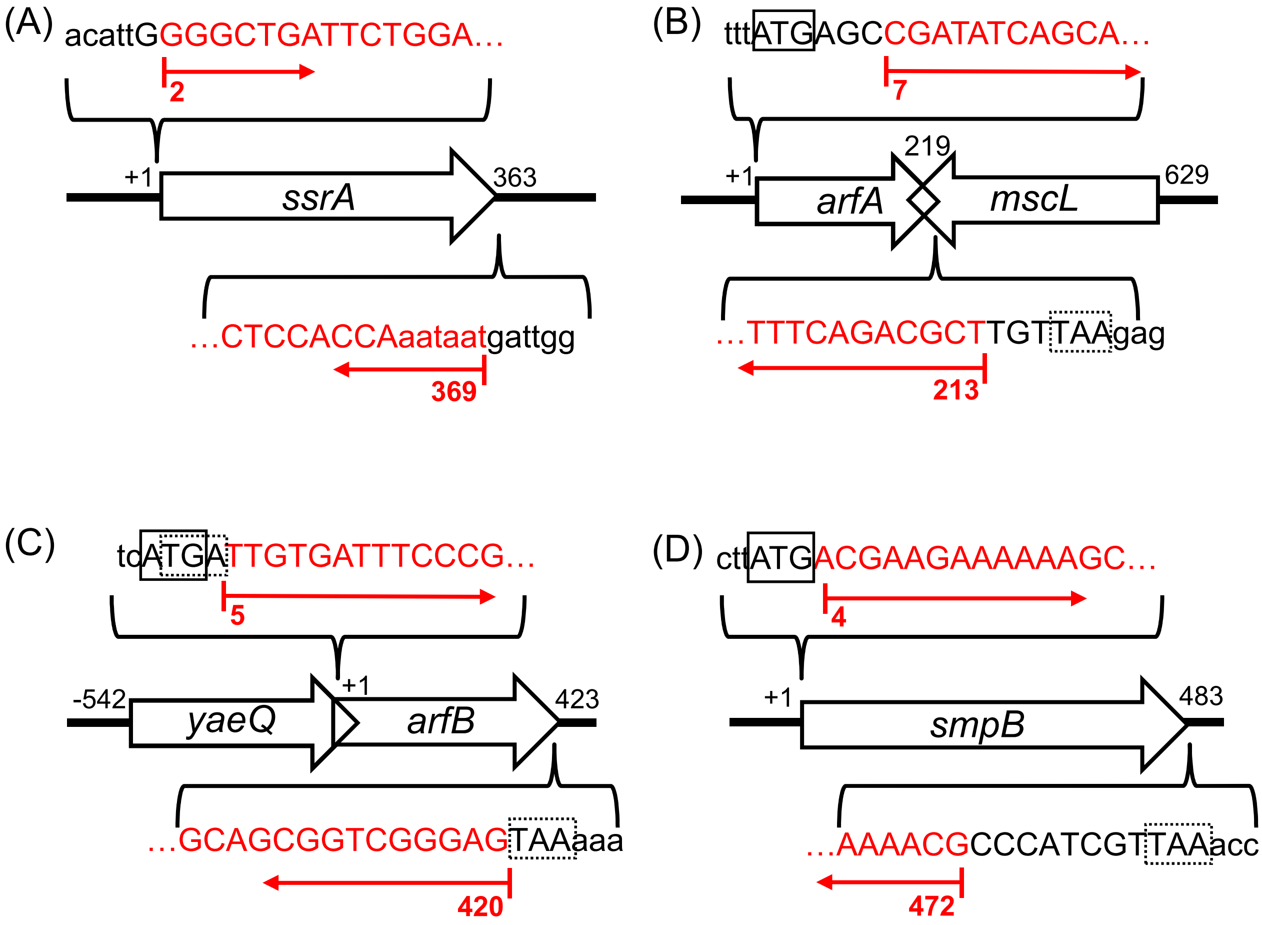

Supplementary Fig. 13. Design for gene deletion of *ssrA*, *arfA*, *arfB*, and *smpB* from SE15 by a suicide vector-mediated genome editing system.

Schematic representations of *ssrA* (A), *arfA* (B), *arfB* (C), and *smpB* (D). Capital letters indicate part of the DNA sequences of *ssrA* and the ORFs of *arfA*, *arfB* and *smpB*. Boxed and dotted boxes indicate the initiation and stop codons, respectively. The +1 position refers to the first nucleotide of *ssrA* (tmRNA) or first one (ATG). Red capital letters as well as red arrows indicate deleted sequences in the genes. The putative polypeptides encoded by the resultant small ORFs of *arfA*, *arfB*, and *smpB* comprised three, eight, and seven amino acid residues, respectively. Notably, *yaeQ* and *arfB* are translationally coupled. Neither the stop codon of *yaeQ* nor *mscL* remains altered by the gene deletions.

Supplementary Table 1. Summary of IC_{50} of antibiotics for *E. coli* SE15.

Antibiotics	Abbr.	IC_{50} µg/mL (µM)
Gentamicin	Gen	2.2 ± 0.2 (1.4 ± 0.1)
Kanamycin	Kan	6.0 ± 1.1 (12 ± 2)
Streptomycin	Str	8.1 ± 0.5 (11 ± 1)
Paromomycin	Par	9.7 ± 0.7 (16 ± 1)
Azithromycin	Azm	1.9 ± 0.2 (2.4 ± 0.3)
Clarithromycin	Clr	32 ± 4 (42 ± 5)
Erythromycin	Ery	19 ± 2 (26 ± 3)
Tetracycline	Tet	0.32 ± 0.04 (0.67 ± 0.08)
Doxycycline	Dox	0.27 ± 0.04 (0.53 ± 0.07)
Oxytetracycline	Otc	0.34 ± 0.01 (0.69 ± 0.01)
Chloramphenicol	Chl	0.71 ± 0.10 (2.2 ± 0.3)
Florfenicol	Ffc	1.4 ± 0.1 (4.0 ± 0.4)
Thiamphenicol	Tap	16 ± 2 (44 ± 6)
Ampicillin	Amp	1.7 ± 0.1 (4.5 ± 0.3)

Data are shown as the mean ± standard deviation of three independent experiments, which were calculated using GraphPad Prism 9.3.1.

Supplementary Table 2. Strains used in this study.

Name	Genotype	Source
SE15	O150:H5, Human commensal strain	Lab stock
SE15 Δ*ssrA*	SE15 Δ*ssrA*	This work
SE15 Δ*arfA*	SE15 Δ*arfA*	This work
SE15 Δ*arfB*	SE15 Δ*arfB*	This work
SE15 Δ*smpB*	SE15 Δ*smpB*	This work
MG1655	F-λ- *ilvG- rfb-50 rph -1*	Lab stock
DH5α	F^- *Φ80lacZ*ΔM15 Δ(*lacZYA-argF*) *U169 recA1 endA1 hsdR17* (r_K^-, m_K^+) *phoA supE44 λ- thi-1 gyrA96 relA1*	Lab stock
SM10λ *pir*	*thi thr leu tonA lacY supE recA*::RP4-2-Tet::Mu Kan^R *pir*	Lab stock

Supplementary Table 3. Plasmids used in this study.

Name	Description	Source
pBR322	pMB1 *ori*, Amp^R, Tet^R	Nippon Gene
pBR322 ΔTet^R	pBR322 ΔTet^R	This study
pArfA	pBR322 P_{arfA}-*arfA*(Δ55-72), ΔTet^R	This study
pArfB	pBR322 P_{arfA}-*arfB*, ΔTet^R	This study
pSsrA/SmpB	pBR322 *smpB-ssrA*, ΔTet^R	This study
pMW118	pSC101 *ori*, par, rep, Amp^R	Nippon Gene
pMW-SsrA	pMW118 *ssrA*(ANDENYALAA)	This study
pMW-$SsrA^{DD}$	pMW118 *ssrA*(ANDENYAL<u>DD</u>)	This study
pABB-CRS2	pR6K γ *ori*, mob RP4, Amp^R, *sacB*, Chl^R	Gift from Prof. Abe, Kitazato Univ.

Supplementary Table 4. Primers used for plasmid construction in this study.

Name	Sequence (5′ → 3′)	Purpose
Del-ssrA(F1)-F	5′- gtcactatggcggccTCCCAGCCTTAGACA CATC -3′	Preparation of *ssrA* deletion strain of SE15
Del-ssrA(F1)-R	5′- gtccttacaccgtccaatcCAATGTGTAACGG TAAGTATACCAG -3′	
Del-ssrA(F2)-F	5′- gattggacggtgtaaggaCTACACCAACAAA AACAGGAAG -3′	
Del-ssrA(F2)-R	5′- taatatttgcccatgAAACGCGCGTAATACT GAC -3′	
Del-arfA(F1)-F	5′- gtcactatggcggccGCCGAAGAACATATC GATC -3′	Preparation of *arfA* deletion strain of SE15
Del-arfA(F1)-R	5′- ccaattggagtttttatgagcTGTTAAGAGCGG TTATTCTGC -3′	
Del-arfA(F2)-F	5′- GCTCATAAAAACTCCAATTGG -3′	
Del-arfA(F2)-R	5′- taatatttgcccatgGGTTACACTCGTATTC TGAAGTG -3′	
Del-arfB(F1)-F	5′- gtcactatggcggccCATCTTTCAGCTCAA GTGTCAG -3′	Preparation of *arfB* deletion strain of SE15
Del-arfB(F1)-R	5′- caccatccattccttcttttaTCATGAAGGTTGTT GCCAG -3′	
Del-arfB(F2)-F	5′- taaaaagaaggaatggatGGTGAAAAAAGC GATAGTGAC -3′	
Del-arfB(F2)-R	5′- taatatttgcccatgGCGAAGGAGACAAAA AGTC -3′	
Del-smpB(F1)-F	5′- gtcactatggcggccACGCTGACCTTTAACA GTAGC -3′	Preparation of *smpB* deletion strain of SE15
Del-smpB(F1)-R	5′- cgcttatataggtttaacgatgggCATAAGCGTCG TGAATCATC -3′	
Del-smpB(F2)-F	5′- cccatcgttaaacctatataAGCGATACTAATCA AATCCTCAC -3′	
Del-smpB(F2)-R	5′- taatatttgcccatgTTCAGAGTTCAACTTAC TGAGTTCC -3′	

ArfA162-F	5′- tcgtcttcaagaattAGCTCGTATAGCCGAG TTG -3′	Preparation of pArfA
ArfA162-R	5′- aagggcatcggtcgaTTAACTGGCCTCCCA GTTC -3′	
ArfB-F	5′- TATGATTGTGATTTCCCGAC -3′	Preparation of pArfB
ArfB-R	5′- TCACCATCCATTCCTTCTT -3′	
Inverse primer-ArfB-F	5′- aggaatggatggtgaGAGCGGTTATTCTGC TCTTTCAGC -3′	
Inverse primer-ArfB-R	5′- gaaatcacaatcataAAAACTCCAATTGGTT ATTTAACGGC -3′	
SmpB-F	5′- ccaattggagtttttATGACGAAGAAAAAA GCACATAAACCTG -3′	Preparation of pSmpB
SmpB-R	5′- atccattccttctttTTAACGATGGGCGTTTT TCATGA -3′	
Inverse primer-SmpB-F	5′- AAAGAAGGAATGGATGGTGAGAG CG -3′	
Inverse primer-SmpB-R	5′- AAAAACTCCAGTTGGTTATTTAAC GGCGCGAGT -3′	
SsrA/SmpB-F	5′- tcacgaggccctttcGCGGTCCGGCTAATC -3′	Preparation of pSsrA/SmpB
SsrA/SmpB-R	5′- ataaccgctctcaccGGTTCGGATTTAA TTAGTTCTCTTC -3′	
Inverse primer-SsrA/SmpB-F	5′- GGTGAGAGCGGTTATTCTG -3′	
Inverse primer-SsrA/SmpB-R	5′- GAAAGGGCCTCGTGATAC -3′	
Check-pBR322-F	5′- CAGGGTTATTGTCTCATGAGC -3′	Check of insertion sequence in pBR322
Check-pBR322-R	5′- ATCGGAGTTACGGACACC -3′	
SsrA-F	5′- gagtcgacctgcaggcatgcATAACGCCA TTGAGGCTG -3′	Preparation of pMW-SsrA

SsrA-R	5′- ggccagtgccaagcttCGCGGGACAAAT TGAG -3′	
$SsrA^{DD}$-F	5′- CGACGAAAACTACGCTTTAGATG ATTAATAACCTGCTTAGAGCCC -3′	Preparation of pMW-$SsrA^{DD}$
$SsrA^{DD}$-R	5′- GGGCTCTAAGCAGGTTATTAATC ATCTAAAGCGTAGTTTTCGTCG -3′	
Check-pMW118-F	5′- GTATCTATCAGTGAAGCATCAAG AC -3′	Check of insertion sequence in pMW118
Check-pMW118-R	5′- ACCCCAGGCTTTACACTTTATG -3′	

Lower-case letters indicate sequence homologs to that in the plasmid for In-Fusion cloning.

Supplementary Table 5. Primers used for qPCR experiments in this study.

Name	Sequence (5′ → 3′)	Target
qPCR-idnT-F	5′- CCTCTTCGCTAAACAGATGA GG -3′	mRNA of *idnT* (Reference Gene)
qPCR-idnT-R	5′- CGACAGTTATTGTCGCAGGA -3′	
qPCR-smpB-F	5′- GGAGAGGCATTTCTGTTTGG -3′	mRNA of *smpB*
qPCR-smpB-R	5′- CGTACAATGAGTCCAGTTCG -3′	
qPCR-ssrA-F	5′- CAAAAGAGATCGCGTGGAAG -3′	tmRNA
qPCR-ssrA-R	5′- GCGTCCGAAATTCCTACATC -3′	
qPCR-arfA-F	5′- CGATAGAAGCATTACTACAT GATCC -3′	mRNA of *arfA*
qPCR-arfA-R	5′- CGCATGTAACTGCCTTTCC -3′	
qPCR-rnc-F	5′- GCTTCAACGGAAGCTG -3′	mRNA of *rnc*
qPCR-rnc-R	5′- GCATTGGCGATAACGTAG -3′	
qPCR-arfB-F	5′- CGGCTATTCATCTGCGTTTT -3′	mRNA of *arfB*
qPCR-arfB-R	5′- CCTGACTGCGGTATTCCTGT -3′	
